# Discriminative Sensing of Structurally Similar Neurotransmitters via In-TBAPy MOF Arrays

**DOI:** 10.3390/nano16140891

**Published:** 2026-07-20

**Authors:** Ting He, Penglei Shen, Hui Xu, Ziyao Zhang, Tao Zhao, Gongxun Bai, Junkuo Gao

**Affiliations:** 1 Key Laboratory of Rare Earth Optoelectronic Materials and Devices of Zhejiang Province, Institute of Optoelectronic Materials and Devices, College of Optical and Electronic Technology, China Jiliang University, Hangzhou 310018, China; 19962643301@163.com (T.H.); shen_penglei@163.com (P.S.); zgjlzzy2001@163.com (Z.Z.); baigx@cjlu.edu.cn (G.B.); 2 Institute of Functional Porous Materials, School of Materials Science and Engineering, Zhejiang Sci-Tech University, Hangzhou 310018, China; 202220301079@mails.zstu.edu.cn

**Keywords:** metal–organic frameworks, In-TBAPy, sensor array, neurotransmitters, pattern recognition, excimer emission

## Abstract

The accurate discrimination of structurally analogous neurotransmitters remains a formidable challenge due to their high structural similarity and overlapping chemical properties. To address the limitations of low specificity in single-probe sensors and the fabrication complexity of multi-component arrays, we developed a simplified fluorescence sensing array based on a single pyrene-functionalized MOF, In-TBAPy. This strategy leverages the distinctive monomer-to-excimer luminescence transition of In-TBAPy, triggered by the tunable π-π stacking of pyrene units within the crystalline framework. The results demonstrate that the array, integrated with Linear Discriminant Analysis (LDA) across four optimized emission channels, achieves a classification accuracy of 93.75% in identifying four highly similar neurotransmitters: serotonin (5-HT), dopamine (DA), adrenaline (A), and norepinephrine (NA). Notably, the sensing platform exhibits exceptional robustness in simulated physiological environments and complex multi-analyte mixtures, enabling reliable quantitative analysis: 0–100 μM for 5-HT and adrenaline (A), 0–40 μM for dopamine (DA), and 0–80 μM for norepinephrine. Mechanistic studies suggest that the differential quenching of monomer and excimer peaks stems from the synergistic effect of competitive absorption and host–guest interactions. This work effectively overcomes the cross-interference issues of traditional sensors and validates a high-efficiency solution for high-throughput neurotransmitter analysis using a single-material-based array strategy, significantly reducing operational costs and preparation time.

## 1. Introduction

**Scheme 1:**
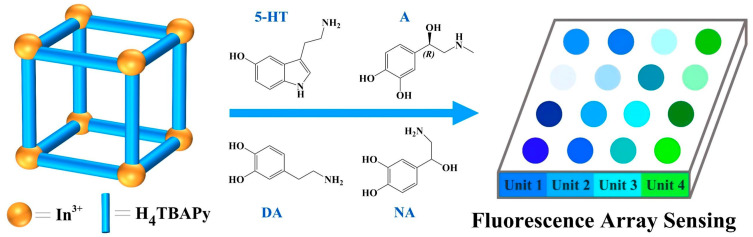
Fluorescent array sensing of four neurotransmitters by In-TBAPy.

Neurotransmitter biomarkers, as key indicators to characterize the pathological state of neurological diseases, are closely related to the disease process with their dynamic changes. For example, for 5-hydroxytryptamine (5-HT), as a monoamine molecule, changes in its level are associated with mood swings, sleep disorders, and eating disorders [1,2]. Dopamine (DA) is the most abundant catecholamine neurotransmitter in the brain, and the abnormal concentration of DA in organisms can lead to a variety of psychiatric disorders such as Alzheimer’s disease and Parkinson’s disease [3,4]. Adrenaline (A) and Noradrenaline (NA) are important neurotransmitters in the sympathetic nervous system, and their abnormal levels have been associated with anxiety disorders, cardiovascular diseases, and other pathological states [5,6,7,8]. By accurately detecting changes in the concentration of these molecules, it can provide an important basis for early diagnosis and personalized treatment. However, as shown in Scheme 1, these four neurotransmitter-based biomarkers are structurally highly similar and tend to interfere with each other during the detection process and drastically reduce the accuracy of the assay. Therefore, highly sensitive and selective immediate monitoring of neurotransmitter-like biomarkers in biological systems is important.

Metal–organic frameworks (MOFs), as a novel class of porous crystalline materials, are constructed by bridging metal ions (clusters) with organic ligands to form a network framework, ultimately resulting in a porous structure through the interaction of various chemical bonds [9,10,11]. The large surface area and porous structure of MOFs provide more interfaces and active sites for interaction between the material and analytes. Additionally, the abundant functional groups of organic ligands make MOFs easily amenable to functionalization with various molecules and materials [12,13,14,15,16,17]. These structural features endow MOFs with significant potential for detecting biomarkers in biological systems. Currently, fluorescence sensors based on MOFs have successfully detected various biomolecules associated with diseases, such as amino acids, DNA, phospholipids, and bilirubin [18,19,20,21,22,23].

Despite this tremendous potential, the dominant design philosophy for these sensors has predominantly followed a “lock-and-key” model, which presents its own set of limitations. While this sensor design effectively addresses the sensitivity issue of detecting analytes, it also exposes several prominent drawbacks. Firstly, the mismatch between interference resistance and material preparation complexity poses a challenge. Simple preparation methods for MOFs-based specific fluorescence sensors are prone to interference from similar substances, leading to a significant decrease in accuracy and reliability [24,25]. Conversely, achieving excellent specificity often requires complex material modification, greatly increasing the difficulty and cost of preparation, which is not conducive to the practical application of luminescent MOFs in production and daily life [26]. On the one hand, the complexity of analytes often requires a vast number of specific fluorescence sensors to be designed if each analyte corresponds to one sensor. Even if a specific fluorescence sensor is designed for each analyte, testing needs to be completed one by one, limiting testing efficiency and making specific fluorescence sensors difficult to adapt to the needs of real-time, in situ testing [27,28]. Therefore, developing high-throughput analysis methods that are more efficient than the “lock-and-key” response model has become a research hotspot.

Array sensors emulate the olfactory system of mammals, comprising numerous sensing units where each unit generates a specific response to the analyte under detection. These responses vary among sensing units. By harnessing the cross-response between sensing units and analytes, array sensors can enhance the identification and discrimination performance of structurally or functionally similar substances, thereby holding significant importance for sensitive and accurate detection of biomarkers within the human body [29,30]. Compared to conventional fluorescence sensors, array fluorescence sensors offer advantages including high throughput, sensitivity, selectivity, and multi-target detection capabilities, enabling simultaneous detection of multiple molecules in a single experiment and providing a more efficient and comprehensive analytical approach for research in the fields of biomedicine and biochemistry [31,32,33]. In recent years, researchers have applied MOFs fluorescence array sensors to the identification and differentiation of metal ions and biological small molecules, and achieved satisfactory results [34,35,36,37,38,39,40]. However, the development of fluorescence array sensing for biomarker-like neurotransmitters is still in its early stages [41,42,43,44]. By precisely controlling the structure of MOFs, multiple non-specific sensing units can be constructed. This leads to the development of MOF-based fluorescence array sensing materials that can simultaneously identify and differentiate similar biomarkers and perform quantitative analysis, which holds significant practical value [45,46].

In this paper, 1,3,6,8-tetrakis (4-carboxy benzene) pyrene (H_4_TBAPy) with pyrene backbone was selected as the organic ligand, and In^3+^ as the metal node, and the porous MOFs with excellent luminescence properties, In-TBAPy, were prepared with the solvent–thermal method. The large π conjugation system of pyrene ring endowed the unique luminescence properties of In-TBAPy, which showed differentiated luminescence in the solid state and aqueous suspension. Using the unique luminescence characteristics of In-TBAPy in aqueous suspension, an In-TBAPy sensing array was constructed with four peak intensities of 440, 455, 475, and 510 nm as the fluorescence sensing units. The results show that the sensing array had differential fluorescence responses to the four neurotransmitter-like biomarkers, 5-HT, DA, A, and NA, and could effectively recognize and differentiate the four neurotransmitters at a concentration of 60 μM, and accurately identify the complex mixtures in the simulated physiological environment of diluted fetal bovine serum. Meanwhile, the quantitative detection of the four neurotransmitter-based biomarkers was realized in the concentration range of 0–100 μM. This study provides a more efficient and comprehensive analytical tool for the development of low-cost and high-throughput neurotransmitter biomarkers.

## 2. Results and Discussion

**Figure 1:**
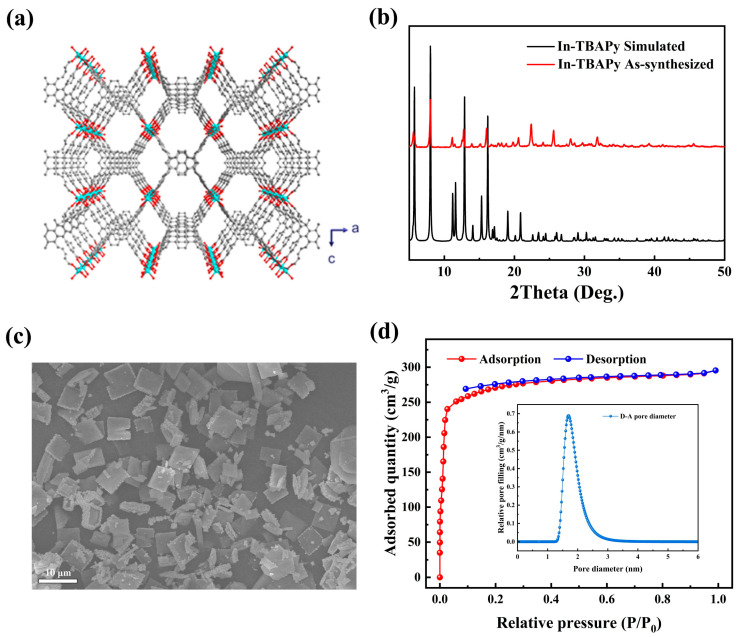
(**a**) Schematic of the porous structure of In-TBAPy; (**b**) PXRD spectra; (**c**) SEM images; (**d**) Nitrogen adsorption and desorption curves and pore size distribution at 77 K.

In this study, a metal–organic framework material containing pyrene, In-TBAPy, was synthesized using a solvothermal method [47]. The framework of the three-dimensional crystal structure of In-TBAPy is constructed through the coordination between In(III) ions and TBAPy ligands (Figure 1a). Specifically, Each In(III) center is coordinated with four TBAPy ligands and two μ_2_-OH (bridging hydroxide ions), forming an InO_4_(OH)_2_ octahedral unit. These octahedral units are connected by TBAPy ligands to form one-dimensional chain structures, which are further bridged by TBAPy ligands to form a three-dimensional open framework. Additionally, the pyrene ring structure of H_4_TBAPy and the 3D pore structure of In-TBAPy along the a-, b-, and c-axes are further detailed (Figure S1). The powder X-ray diffraction (PXRD) pattern of the synthesized In-TBAPy powder matches perfectly with the simulated diffraction peaks derived from the ideal single-crystal data, indicating that the synthesized In-TBAPy exhibits the expected crystal structure and high phase purity (Figure 1b). Furthermore, scanning electron microscopy (SEM) observations reveal that the sample exhibits a well-defined cubic morphology with a uniform size of several μm (Figure 1c). EDS elemental mapping was performed to further verify the uniform elemental distribution of the as-synthesized In-TBAPy crystalline material (Figure S2). The homogeneous co-distribution of In, C and O across the micrometer scale without element segregation or aggregation confirms the single-phase integral framework of In-TBAPy.

MOFs possess a large specific surface area and a tunable pore structure, which enables them to adsorb and store target molecules efficiently, a property that is extremely important in fluorescence sensing applications as it allows for efficient interactions between MOFs and target molecules. Nitrogen adsorption–desorption isotherms at 77 K confirmed the highly ordered microporous structure of In-TBAPy, revealing a high specific surface area of 961.4 m^2^·g^−1^ and an average pore size of 18 Å (Figure 1d). The large and regular three-dimensional pore structure of In-TBAPy, with its own high specific surface area, provides a highly efficient detection efficiency in fluorescence sensing of 5-HT, DA, A, and NA. Thermogravimetric analysis (TGA) was performed to evaluate the thermal stability of In-TBAPy. The framework remains stable up to approximately 400 °C (Figure S3), indicating the high thermal stability of the In-TBAPy.

**Figure 2:**
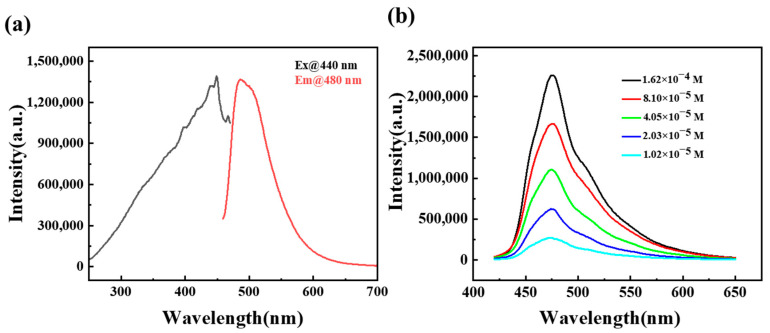
Fluorescence spectra of In-TBAPy in (**a**) solid state and (**b**) different concentrations of deionized water suspension.

In metal–organic frameworks (MOFs), fluorescence properties may be induced by specific functional groups or ligands within the framework. These properties can change due to environmental conditions (such as temperature, pressure, pH) or interactions with other substances (such as adsorbed gas or solvent molecules). To explore this, the photoluminescence properties of In-TBAPy were systematically investigated in both the solid state and aqueous suspensions at various concentrations. Upon excitation at 440 nm, the solid-state In-TBAPy powder exhibits a main fluorescence emission peak at 487 nm accompanied by a shoulder peak at 508 nm (Figure 2a). While under 380 nm excitation, the fluorescence spectra of the In-TBAPy aqueous suspensions display a broader emission profile, featuring a main peak at 475 nm and two shoulder peaks at 455 nm and 510 nm (Figure 2b). For fluorescent molecules with large π-conjugated structures such as pyrene rings, the distance between π-conjugated rings and the parallel stacking staggering angle affects the luminescence. Therefore, the different luminescent forms of In-TBAPy can be attributed to the π-π interactions of pyrene cores, where the pyrene cores in the solid state assembles through strong π-π interactions, leading to red-shifted excimer luminescence, while in the aqueous suspension, the MOF shows monomer luminescence due to solvent molecular interactions. Meanwhile, it can be observed that the luminescence intensity increases with the increase in suspension concentration. Therefore, the suspension at a concentration of 1.62 × 10^−4^ M (which exhibits optimal luminescence intensity) was selected for the subsequent fluorescence sensing experiments.

To determine the potential of In-TBAPy as a sensor array for neurotransmitter molecules, we selected four luminescent units of In-TBAPy, I-440 nm, I-455 nm, I-475 nm, and I-510 nm, to measure the fluorescence response of In-TBAPy to four neurotransmitter biomarkers: 5-HT, DA, A, and NA. Among them, I-455 nm, I-475 nm, and I-510 nm are the inherent emissions of the In-TBAPy deionized water suspension, and I-440 nm is a new luminescent unit that appears after the addition of some analytes. As shown in Figure S4, the fluorescence emission peak ratio curves and fluorescence response spectra of In-TBAPy in deionized water suspension to different concentrations of analytes (0–100 μM) indicate that as the concentration of the analytes change, In-TBAPy exhibits significant fluorescence quenching or enhancement, showing the following differentiated characteristics: (1) 5-HT shows significant fluorescence quenching for all four emission bands of I-440 nm, I-455 nm, I-475 nm, and I-510 nm (Figure S2a,b); (2) DA shows fluorescence enhancement at I-440 nm and slight quenching at I-455 nm, I-475 nm, and I-510 nm (Figure S2c,d); (3) A shows significant enhancement at I-440 nm, no significant effect at I-455 nm, and slight quenching at I-475 nm and I-510 nm (Figure S2e,f); (4) NA shows the largest enhancement in fluorescence intensity at I-440 nm, followed by I-455 nm, with slight enhancement at I-475 nm, and no significant change at I-510 nm (Figure S2g,h).

In addition to changes in fluorescence intensity, the corresponding fluorescence color also showed differential changes. As shown in the inset of Figure S2, before the addition of the analyte, the luminescence of In-TBAPy was a cyan emission located at 475 nm, and with the increase of 5-HT, the luminescence of In-TBAPy was gradually quenched and the luminescence intensity was decreased, but the luminescence color did not change in a relatively obvious way (Figure S2b). After adding DA, the luminescence color of In-TBAPy gradually changed from cyan to blue, but the intensity was slightly reduced (Figure S2d). After adding NA, the luminescence color of In-TBAPy changed from cyan to light blue, and the luminescence color of In-TBAPy changed to dark blue with high concentration of NA, and the brightness first decreased and then increased (Figure S2f). The luminescence color of In-TBAPy changed from cyan to light blue to dark blue with a gradual increase in intensity after the addition of A (Figure S2h). In-TBAPy showed a dual differential response of fluorescence color and intensity to the four neurotransmitter biomarkers.

Four sensing channels at 440, 455, 475, and 510 nm were selected based on the intrinsic fluorescence spectrum of In-TBAPy. The 455, 475, and 510 nm peaks correspond to monomer and excimer emissions, while the 440 nm band is analyte-induced. Channel stability was verified across three independent batches and three days of storage (Figures S5–S8). Peak positions remained unchanged in all cases, with only slight intensity attenuation. After clarifying that the different sensing units in In-TBAPy showed differential fluorescence responses to the four neurotransmitter biomarkers, linear discriminant analysis, a statistical analysis tool, was used to determine the recognition and differentiation performance of the fluorescence array sensor consisting of the above four sensing units for the four analytes.

**Figure 3:**
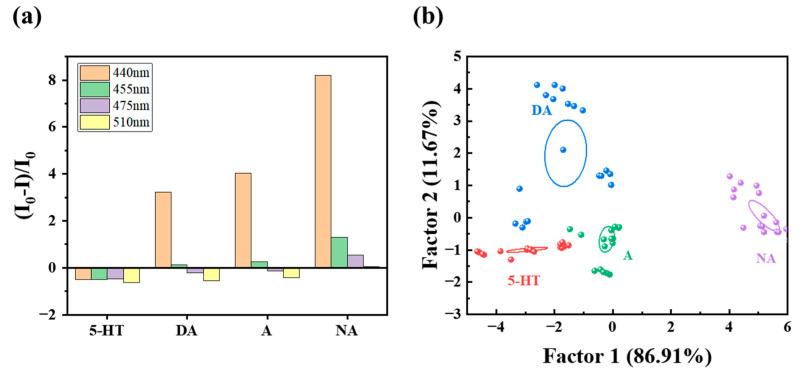
(**a**) Fluorescence response fingerprints of the four sensing units of In-TBAPy in deionized water in response to a concentration of 60 μM of DA, 5-HT, A, and NA; (**b**) Typical plots obtained after linear discriminant analysis.

The concentration of each neurotransmitter was fixed at 60 μM, and the fluorescence emission spectra of In-TBAPy were repeated five times before and after the addition of each analyte, and the fluorescence intensities of the four sensing units at I-440 nm, I-455 nm, I-475 nm, and I-510 nm were recorded. A total of 20 training samples were obtained, along with the characteristic fingerprints of each analyte at a concentration of 60 μM (Figure 3a). Immediately after that, the 80 training samples were divided into four groups according to the type of analyte and analyzed by LDA, resulting in the typical plot shown in Figure 3b. As shown in this figure, the first two factors (Factor 1 = 86.91%, Factor 2 = 11.67%) obtained from the LDA accounted for as much as 98.58% of the total variance, which exceeded the 95% threshold, which is sufficient to prove that the first two factors can be used to achieve the distinction between classes; in the two-dimensional typical plot, all analytes have clear and explicit boundaries, and there is no overlap. In the two-dimensional typical plot, all analytes are clearly defined, and there is no overlapping, which indicates that the fluorescent array sensor composed of four sensing units, I-440 nm, I-455 nm, I-475 nm, and I-510 nm, can accurately distinguish four structurally similar neurotransmitter biomarkers.

To further verify the recognition capability of the fluorescent sensor array toward the four neurotransmitter biomarkers, an independent blind test was performed. The analyte concentration was fixed at 60 μM, and 80 unknown blind samples (20 samples per analyte) were independently prepared following the same experimental protocol as the training samples. The fluorescence response data from these blind samples were then classified using the LDA model established from the training dataset. As a result, 75 of the 80 blind samples were correctly classified, corresponding to a prediction accuracy of 93.75% (Table S1). These results demonstrate the excellent predictive capability and robust discrimination performance of the In-TBAPy-based fluorescent sensor array toward these four structurally similar neurotransmitter biomarkers. A comparison of the sensing performance of our In-TBAPy array with other reported MOF-based sensors (Table S3) reveals that our platform offers distinct advantages in simultaneous discrimination of structurally similar neurotransmitters with a simple, label-free, and high-throughput fluorescence readout [48,49,50,51,52,53,54].

In addition, the sensitivity of the sensor to the analyte to be tested can be measured by the limit of detection (LOD) for each analyte. The concentration-dependent fluorescence response curves of In-TBAPy toward the four neurotransmitters exhibited excellent linearity (Figure S9). Specifically, the calibration curve for 5-HT was fitted using the emission intensity at 475 nm, whereas those for DA, A, and NA were fitted using the intensity at 440 nm. Finally, the detection limits of the four neurotransmitter-based biomarkers were calculated to 2.26 μM, 10.13 μM, 3.50 μM and 3.36 μM, respectively.

**Figure 4:**
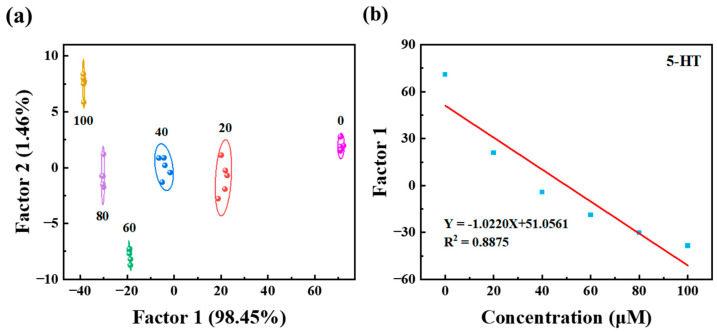
Fluorescence sensing array for 5-HT quantification (**a**) typical plot, (**b**) linear relationship.

In the study of fluorescent sensors, in addition to their performance in similarity recognition and differentiation, the quantitative detection ability is also highly regarded. To explore the possibility of the array sensor for quantitative analysis of multiple neurotransmitter substances, the fluorescence emission spectra of In-TBAPy were tested at six concentrations of each neurotransmitter (0, 20, 40, 60, 80, and 100 μM). For each analyte, a total of 30 independent sample data points were collected and categorized into six groups based on their concentrations. Subsequent linear discriminant analysis (LDA) generated well-defined canonical score plots (Figure 4a and Figure S4a,c,e). It can be seen that in all typical diagrams, the boundaries between different solubility classifications of each analyte molecule are very clear, with no overlapping parts. Therefore, the first canonical factor was utilized as the primary parameter for the quantitative detection of each neurotransmitter (Figure 4b and Figure S4b,d,f). In all tested four neurotransmitter biomarkers, the changes in fluorescence emission intensity of 5-HT and A in the concentration range of 0–100 μM are well correlated with the first typical factor, the changes in fluorescence emission intensity of DA in the concentration range of 0–40 μM are well correlated with the first typical factor, and the changes in fluorescence emission intensity of NA in the concentration range of 0–80 μM are well correlated with the first typical factor, and the linear correlation coefficients of the analytes are all above 0.95, thereby proving that the In-TBAPy fluorescence array sensor has potential quantitative analysis ability for four types of neurotransmitter biomarkers within a certain concentration range.

**Figure 5:**
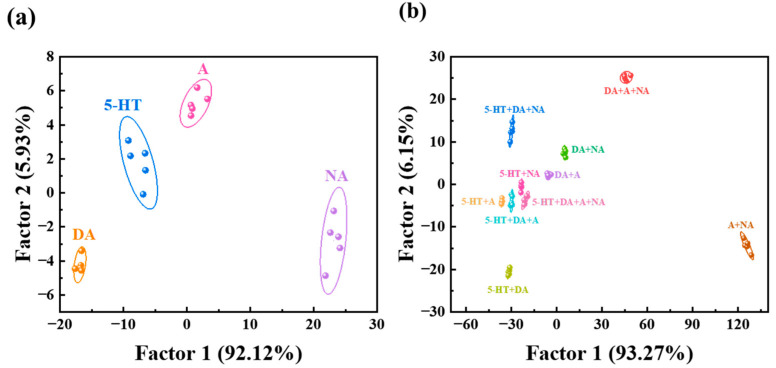
(**a**) Typical plots of the fluorescence response intensities of In-TBAPy in serum to DA, 5-HT, A and NA at a concentration of 60 μM obtained by linear discriminant analysis; (**b**) Typical plots of the fluorescence response intensities of multivariate mixtures of DA, 5-HT, A and NA at a total concentration of 60 μM obtained by linear discriminant analysis.

Although In-TBAPy has good discriminatory performance for four neurotransmitter biomarkers, the real testing environment is often complex. For this reason, this study designed two sets of validation experiments to assess its anti-interference performance. Firstly, the physiological environment was simulated to explore the recognition ability of In-TBAPy in serum for four neurotransmitter biomarkers. Fetal bovine serum was diluted 30-fold and then In-TBAPy was added to prepare a suspension, and the rest of the test and analysis steps maintained the experimental parameter settings consistent with the previous assay process. The canonical score plot reveals clearly separated boundaries among the four categories (Figure 5a), indicating that the complex serum matrix did not significantly interfere with the sensing signals and demonstrating the sensor’s robust resistance to environmental disturbances. In addition, a multivariate mixture of four neurotransmitter biomarkers with a total concentration of 60 μM was targeted for identification and differentiation. Furthermore, mixed samples with varying combination ratios formed distinct, independent clusters within the discrimination space without any overlapping (Figure 5b). The successful differentiation of these diverse mixtures demonstrates that the In-TBAPy sensor can effectively resist mutual interference among structurally similar substances. To further evaluate the selectivity of the sensor, we examined its fluorescence response towards various interfering substances, including inorganic salts (KCl, MgCl_2_, NaCl, CaCl_2_), amino acids (glycine, proline), glucose, and urea. Barely any obvious variation in fluorescence emission was observed after introducing these interferents, which demonstrates the outstanding selectivity of the In-TBAPy-based sensor array (Figure S10). The results confirm the immense potential of the In-TBAPy array sensor for practical applications in complex environments.

To assess practical usability, we characterized batch consistency and short-term storage performance of In-TBAPy (Figures S5–S8). Three identically synthesized batches show nearly overlapping aqueous fluorescence and consistent sensing spectra toward four neurotransmitters, confirming good repeatability. When sealed and stored for 1–3 days, the dispersed In-TBAPy suspension maintains unchanged emission peaks and intact analyte fluorescence fingerprints with only slight signal attenuation, demonstrating stable sensing performance over at least three days.

In addition, the fluorescence detection mechanism of In-TBAPy for four neurotransmitter biomarkers was also investigated. The PXRD of In-TBAPy after immersion in different analyte solutions exhibited slightly weakened diffraction peak intensities, but the peak positions remained fundamentally unchanged (Figure S12), indicating that the addition of the analytes did not cause obvious change in the structure of the material. To further elucidate the nature of the host–guest interactions, we compared the FT-IR spectra of In-TBAPy before and after neurotransmitter adsorption (Figure S13). At representative vibrational regions, the spectra remained essentially unchanged, with no obvious peak shifts or line shape variations. In particular, the invariant carboxylate stretching vibrations indicate that no coordinative or covalent bonding occurs between the neurotransmitters and the In^3+^ nodes, while the unchanged pyrene C=C vibration demonstrates that the pyrene chromophores remain chemically intact during the sensing process. These observations support a sensing mechanism dominated by non-covalent host-guest interactions. Furthermore, the preserved FT-IR fingerprint confirms the structural stability of the framework throughout the sensing process. Combined with the UV-vis spectral overlap discussed above, these results provide experimental support for the proposed mechanism involving non-covalent host–guest interactions and competitive absorption. Spectral analysis revealed a significant overlap between the excitation spectrum of In-TBAPy and the absorption spectra of the analytes, with 5-HT displaying the maximum overlap (Figure S14). This result suggests a strong competitive absorption mechanism between 5-HT and the MOF, which effectively drives the severe fluorescence quenching observed following the introduction of 5-HT. The fluorescence lifetime decay curve of In-TBAPy at the original 475 nm emission was first recorded (Figure S15). Subsequently, the fluorescence lifetime decay curve of In-TBAPy at 440 nm was also recorded (Figure S16). The fluorescence lifetime of the pristine In-TBAPy sample at 440 nm was 16.32 ns via bi-exponential fitting. In contrast, the decay curves after the addition of 5-HT, DA, A, and NA were well described by mono-exponential fitting, with the lifetimes of 1.51 ns, 2.30 ns, 2.48 ns, and 2.31 ns, respectively (Figure S17). These distinct lifetime changes reveal differentiated interactions between analytes and In-TBAPy.

In addition, the 3D pore sizes of In-TBAPy were 0.60 × 0.29 nm (along the [100] crystal direction), 0.49 × 0.98 nm (along the [010] crystal direction), 0.90 × 0.68 nm (along the [010] crystal direction), and 0.48 × 0.37 nm (along the [001] crystal direction), whereas the 3D dimensions of 5-HT, DA, A, and NA were 1.19 × 0.83 × 0.40 nm, 1.08 × 0.74 × 0.40 nm, 1.07 × 0.69 × 0.69 nm, and 1.00 × 0.70 × 0.63 nm, respectively, and the analytes were smaller than the largest pore of the MOF. Thus, all four neurotransmitters could enter into In-TBAPy pore. Meanwhile, it is observed that the structures of DA, A, and NA all have benzene rings, and after entering the pore of In-TBAPy, their aromatic systems and pyrene groups of the framework ligands change the original electron cloud distribution of the ligands through the π-π stacking interaction, which in turn generates a new excited state radiative excursion pathway, leading to the emergence of new peaks at 440 nm and differential fluorescence responses. To quantify analyte-framework interactions, two fitting models were applied to titration curves (Figures S18–S21). 5-HT yields a large K_SV_ = 1.506 × 10^4^ M^−1^ via Stern-Volmer fitting, and this relatively high value reveals a strong interaction between 5-HT and the framework, which originates from π–π interactions. For DA, A and NA, Benesi-Hildebrand fitting gives apparent K_d_. Since a smaller K_d_ value indicates stronger host-guest interaction, the interaction strength follows the order A > NA > DA. Compared with 5-HT, these catecholamines show weaker and more reversible interactions with the framework, which mainly perturb the electronic environment of the pyrene-based ligands and promote the emergence of a new emission band at 440 nm rather than causing overall fluorescence quenching. These quantitative results further explain the different luminescence responses of In-TBAPy toward 5-HT and catecholamine analytes. In summary, the preliminary analysis suggests that the mechanism can be attributed to the differentiated interactions and competitive absorption effects between analytes and materials.

## 3. Conclusions

In this paper, a porous metal–organic framework (MOF) In-TBAPy fluorescent array sensor based on the pyrene derivative H_4_TBAPy was successfully constructed. The sensor integrated multiple sensing array units into a single MOF matrix, realizing efficient recognition and quantitative detection of four neurotransmitter-like biomarkers, namely 5-HT, DA, A, and NA. By precisely modulating the π-π stacking pattern of pyrene-based ligands and the coordination microenvironment of In^3+^ nodes, the material exhibits an emission spectrum that can be switched between solid and aqueous suspensions, forming a sensing unit with four characteristic peaks at 440 nm, 455 nm, 475 nm, and 510 nm. The sensing array breaks through the limitations of the traditional “lock-and-key” model, and utilizes the non-covalent interaction and competitive absorption effects of the differences between the analytes and the materials to achieve precise differentiation of structurally similar neurotransmitters, and the accuracy of the linear discriminant analysis reaches 93.75%. In particular, the sensor maintains excellent anti-interference performance in diluted serum environment and is able to analyze the complex mixtures of four neurotransmitters. Meanwhile, quantitative detection of the four neurotransmitter biomarkers was achieved with separate linear ranges: 0–100 μM for 5-HT and A, 0–40 μM for DA, and 0–80 μM for NA. This work not only provides an effective and high-throughput tool for neurotransmitter analysis but also opens new avenues for designing intelligent MOF-based sensor arrays for complex biosensing applications, such as point-of-care diagnostics for neurological disorders.

## Data Availability

The data that support the findings of this study are available from the corresponding author upon reasonable request.

## Supplementary Materials

The following supporting information can be downloaded at: https://www.mdpi.com/article/10.3390/nano16140891/s1. The experimental details are provided in the Supporting Information. Experimental details including materials, synthesis, fluorescence sensing experiments, limit of detection (LOD), linear discriminant analysis (LDA), and quantitative fitting methods; characterization data including EDX elemental mapping, TGA, PXRD, FT-IR, UV–vis absorption, fluorescence spectra, fluorescence lifetime, and fitting curves (Figures S1–S21); blind test results, confusion matrix, and comparison with reported MOF-based sensors (Tables S1–S3).

## References

[1] Bruce K.R., Steiger H., Koerner N., Israel M., Young S. (2004). Bulimia nervosa with co-morbid avoidant personality disorder: Behavioural characteristics and serotonergic function. Psychol. Med..

[2] Pivac N., Mück-Šeler D., Barišić I., Jakovljević M., Puretić Z. (2001). Platelet serotonin concentration in dialysis patients with somatic symptoms of depression. Life Sci..

[3] Stellar J.R., Kelley A.E., Corbett D. (1983). Effects of peripheral and central dopamine blockade on lateral hypothalamic self-stimulation: Evidence for both reward and motor deficits. Pharmacol. Biochem. Behav..

[4] Chowdhury S., Nugraha A.S., O’May R., Wang X., Cheng P., Xin R., Osman S.M., Hossain M.S., Yamauchi Y., Masud M.K. (2024). Bimetallic metal-organic framework-derived porous one-dimensional carbon materials for electrochemical sensing of dopamine. Chem. Eng. J..

[5] Berecek K.H., Brody M.J. (1982). Evidence for a neurotransmitter role for epinephrine derived from the adrenal medulla. Am. J. Physiol.-Heart Circ. Physiol..

[6] Von Euler U.S. (1971). Adrenergic neurotransmitter functions. Science.

[7] Axelrod J. (1971). Noradrenaline: Fate and control of its biosynthesis. Science.

[8] Kelly C.B., Cooper S.J. (2001). Noradrenaline: The forgotten neurotransmitter. Ir. J. Psychol. Med..

[9] Xu K., Zhang S., Zhuang X., Zhang G., Tang Y., Pang H. (2024). Recent progress of MOF-functionalized nanocomposites: From structure to properties. Adv. Colloid Interface Sci..

[10] Li J.-R., Kuppler R.J., Zhou H.-C. (2009). Selective gas adsorption and separation in metal–organic frameworks. Chem. Soc. Rev..

[11] Li X., Chen K., Guo R., Wei Z. (2023). Ionic liquids functionalized MOFs for adsorption. Chem. Rev..

[12] Jo Y.M., Jo Y.K., Lee J.H., Jang H.W., Hwang I.S., Yoo D.J. (2023). MOF-based chemiresistive gas sensors: Toward new functionalities. Adv. Mater..

[13] Zhang Y., Yin B.H., Huang L., Ding L., Lei S., Telfer S.G., Caro J., Wang H. (2025). MOF membranes for gas separations. Prog. Mater. Sci..

[14] Zhang L., Wang G.-D., Zhang B., Yang G.-P., Zhang W.-Y., Hou L., Wang Y.-Y., Zhu Z. (2024). Creating a MOF with larger pores and higher stability for gas separation through continuous structure transformation. J. Mater. Chem. A.

[15] Payam A.F., Khalil S., Chakrabarti S. (2024). Synthesis and characterization of MOF-derived structures: Recent advances and future perspectives. Small.

[16] Sun N., Shah S.S.A., Lin Z., Zheng Y.-Z., Jiao L., Jiang H.-L. (2025). MOF-based electrocatalysts: An overview from the perspective of structural design. Chem. Rev..

[17] Dolgopolova E.A., Rice A.M., Martin C.R., Shustova N.B. (2018). Photochemistry and photophysics of MOFs: Steps towards MOF-based sensing enhancements. Chem. Soc. Rev..

[18] Yan B. (2017). Lanthanide-functionalized metal–organic framework hybrid systems to create multiple luminescent centers for chemical sensing. Acc. Chem. Res..

[19] Hu Z., Deibert B.J., Li J. (2014). Luminescent metal–organic frameworks for chemical sensing and explosive detection. Chem. Soc. Rev..

[20] Wang J., Li D., Ye Y., Qiu Y., Liu J., Huang L., Liang B., Chen B. (2021). A fluorescent metal–organic framework for food real-time visual monitoring. Adv. Mater..

[21] Wu S., Min H., Shi W., Cheng P. (2020). Multicenter metal–organic framework-based ratiometric fluorescent sensors. Adv. Mater..

[22] Mao Y., Xiong R., Tian J., Ling G., Zhang P. (2025). Advances and applications of metal–organic framework/molecularly imprinted polymer (MOF/MIP) for fluorescence detection. Coord. Chem. Rev..

[23] Yu L., Feng L., Wei Z., Wang S., Feng Y., Shen Y., Cai J., Wu J., Xiao Y. (2023). Tunable Fluorescent Artificial Receptor Biosensor Based on Programmable Lanthanide Metal-Organnic Framework for Highly Selective Neurotransmitter Detection. Adv. Funct. Mater..

[24] Shi W., Zhang S., Wang Y., Xue Y., Chen M. (2022). Preparation of dual-ligands Eu-MOF nanorods with dual fluorescence emissions for highly sensitive and selective ratiometric/visual fluorescence sensing phosphate. Sens. Actuators B Chem..

[25] Chen Q., Li S., Tu X., Zhang X. (2024). Skin-attachable Tb-MOF ratio fluorescent sensor for real-time detection of human sweat pH. Biosens. Bioelectron..

[26] Wang M., Han Y., Sun G., Chen J., Zhao X., Qiu K., Lu S., Liu D., Wang S., Wang H. (2025). Polyfluoroalkyl Substance-Induced Nanoclusters Immobilized MOF-on-MOF Architecture Dissociation-Driven Machine Learning-Assisted Ratio Fluorescence Sensor Array. Anal. Chem..

[27] Pal T.K. (2023). Metal–organic framework (MOF)-based fluorescence “turn-on” sensors. Mater. Chem. Front..

[28] Yu Z., Guo H., Yang Z., Wang M., Yang W. (2024). An ultrasensitive and visual ratio fluorescence sensing platform for tannic acid in water based on boric acid functionalized lanthanide MOF. J. Environ. Chem. Eng..

[29] Bushdid C., Magnasco M.O., Vosshall L.B., Keller A. (2014). Humans can discriminate more than 1 trillion olfactory stimuli. Science.

[30] Li Z., Askim J.R., Suslick K.S. (2018). The optoelectronic nose: Colorimetric and fluorometric sensor arrays. Chem. Rev..

[31] Mao H., Yu L., Tu M., Wang S., Zhao J., Zhang H., Cao Y. (2024). Recent advances on the metal-organic frameworks-based biosensing methods for cancer biomarkers detection. Crit. Rev. Anal. Chem..

[32] Zhang W., You S., Sun J., Lv Y., Wang X., Li X., Su Z. (2025). Multi-emission fluorescent array sensors based on metal-organic frameworks: Recent research progress in synthesis strategies and applications. Coord. Chem. Rev..

[33] Grover K., Waseem M.T., Guo H., New E.J. (2025). Quantitative Analysis of Fluorescent Sensor Arrays. Anal. Sens..

[34] Yan Z., Cai Y., Zhang J., Zhao Y. (2022). Fluorescent sensor arrays for metal ions detection: A review. Measurement.

[35] Zhang W., Sun J., Li X., Wang S., Zhang W., Gong Y., Liu L., Su Z. (2025). Lanthanide MOF-based luminescent sensor array for detection and identification of contaminants in water and biomarkers. Talanta.

[36] Chen B., Mo X., Qu X., Xu Z., Zheng S., Fu H. (2024). Multiple-emitting luminescent metal–organic framework as an array-on-a-MOF for rapid screening and discrimination of nitroaromatics. Anal. Chem..

[37] Zhang R., Yu X., Sun Y., Su C., Wang T., Yu J., Niu N., Chen L., Ding L. (2024). A rapid and accurate fluorescent sensor array based on lanthanide metal-organic framework for identification and determination of perfluorinated compounds. Talanta.

[38] Xu X., Wang X., Ding Y., Zhou X., Ding Y. (2024). Integration of lanthanide MOFs/methylcellulose-based fluorescent sensor arrays and deep learning for fish freshness monitoring. Int. J. Biol. Macromol..

[39] Ran H., Tang Y., Wu Z., Zhou J., Tao H., Wu Y. (2024). A colorimetric sensor array based on bimetallic CeCo-MOF with triple-enzyme-mimic activities for highly sensitive detection of tetracycline antibiotics. Chem. Eng. J..

[40] Tang K., Zuo W., Tan Y., Wang X., Zhou Q., Zhang Z. (2025). Microvolume ratiometric fluorescence capillary imprinted array sensor based on bimetallic-nanozyme for rapid intelligent visual detection of five glucocorticoids. Sens. Actuators B Chem..

[41] Wang X., Gopalsamy K., Clavier G., Maurin G., Ding B., Tissot A., Serre C. (2024). Lanthanide MOF-based luminescent sensor arrays for the detection of castration-resistant prostate cancer curing drugs and biomarkers. Chem. Sci..

[42] Rana A., Mishra G., Biswas S. (2024). Functional group-assisted fluorescence sensing platform for nanomolar-level detection of an antineoplastic drug and a neurotransmitter from environmental water and human biofluids. Inorg. Chem..

[43] Liu L., Ru L., Tang H., Zhang Z., Feng W. (2023). A multi-responsive Tb-doped MOF probe for highly specific breath volatile biomarker recognition of lung cancer. J. Mater. Chem. C.

[44] Yu K., Lu S., Qiu K., Zhang Y., Sun A., Gong S., Wang K., Gao X., Xu X., Wang H. (2025). Biomimetic Analysis of Neurotransmitters for Disease Diagnosis through Light-Driven Nanozyme Sensor Array and Machine Learning. Adv. Sci..

[45] Li T., Zhu X., Hai X., Bi S., Zhang X. (2023). Recent progress in sensor arrays: From construction principles of sensing elements to applications. ACS Sens..

[46] Yu X., Fu L., Wang T., Liu Z., Niu N., Chen L. (2024). Multivariate chemical analysis: From sensors to sensor arrays. Chin. Chem. Lett..

[47] Stylianou K.C., Heck R., Chong S.Y., Bacsa J., Jones J.T.A., Khimyak Y.Z., Bradshaw D., Rosseinsky M.J. (2010). A guest-responsive fluorescent 3D microporous metal-organic framework derived from a long-lifetime pyrene core. J. Am. Chem. Soc..

[48] Zhang W., Zhang Z., Bai G., Xu S., Xu H., Gao J. (2026). Excimer-to-monomer switching in a pyrene HOF for discriminative neurotransmitter sensing. Chem. Commun..

[49] Chen F., Cai Y., Shen P., Bai G., Alshahrani T., Gao J., Chen B., Xu S., Xu H. (2023). A porous hydrogen-bonded organic framework for sensitive and highly selective fluorescence sensing of carcinoid biomarkers. Microporous Mesoporous Mater..

[50] Jiang Z., Zheng H.-Q., Guan L., Yang Y., Cui Y., Qian G. (2022). Enhanced luminescence in multivariate metal–organic frameworks through an isolated-ligand strategy. J. Mater. Chem. C.

[51] Dai L.-M., Li L., Wang E.-L., You S.-Y., Zhang L., Gu Z., Wu M.-F., Zou J.-Y. (2025). Encapsulation of Fluorophores within the Lanthanide Metal–Organic Framework for Regulating the Ratiometric Dynamic Luminescence Performance of Serotonin Detection. Inorg. Chem..

[52] Wu K.-Y., Chen M., Huang N.-H., Li R.-T., Pan W.-L., Zhang W.-H., Chen W.-H., Chen J.-X. (2021). Facile and recyclable dopamine sensing by a label-free terbium(III) metal-organic framework. Talanta.

[53] Liu Y., Xu X., Yan B. (2022). An anthracene-based hydrogen-bonded organic framework as a bifunctional fluorescent sensor for the detection of γ-aminobutyric acid and nitrofurazone. Inorg. Chem. Front..

[54] Wang N., Xie M., Wang M., Li Z., Su X. (2020). UiO-66-NH_2_ MOF-based ratiometric fluorescent probe for the detection of dopamine and reduced glutathione. Talanta.

