# Supplementary material for: Discriminative Sensing of Structurally Similar Neurotransmitters via In-TBAPy MOF Arrays
Source: Nanomaterials (Basel). 2026 Jul 20;16(14):891. doi: 10.3390/nano16140891 (PMC13415075; doi:10.3390/nano16140891)
Supplement: Supplementary file 1 [file nanomaterials-16-00891-s001.zip › nanomaterials-4367308-supplementary.pdf]

Supplementary Materials

Discriminative Sensing of Structurally Similar Neurotransmitters via In-TBAPy MOF Arrays

Ting He [1,†], Penglei Shen [1,†], Hui Xu [1,*], Ziyao Zhang [1], Tao Zhao [2], Gongxun Bai [1] and Junkuo Gao [2,*]

[1] Key Laboratory of Rare Earth Optoelectronic Materials and Devices of Zhejiang Province, Institute of Optoelectronic Materials and Devices, College of Optical and Electronic Technology, China Jiliang University, Hangzhou 310018, China; 19962643301@163.com (T.H.); shen_penglei@163.com (P.S.); zgjlzzy2001@163.com (Z.Z.); baigx@cjlu.edu.cn (G.B.)

[2] Institute of Functional Porous Materials, School of Materials Science and Engineering, Zhejiang Sci-Tech University, Hangzhou 310018, China; 202220301079@mails.zstu.edu.cn

* Correspondence: huixu@cjlu.edu.cn (H.X.); jkgao@zstu.edu.cn (J.G.)

† These authors contributed equally to this work.

1. Experimental Section

1.1. Materials and general methods

All chemicals were purchased commercially without further purification. The powder X-ray diffraction (PXRD) images were measured on D2 PHASER diffractometer at a scan rate of 5 °/min (5 °– 50 ° of scan range) via Cu Kα radiation (λ = 1.542 Å). The Field Emission Scanning Electron Microscopy (FE-SEM) images were carried out on MODEL SU8010. The fluorescence detection experiments and the PL decay curves were carried out on HORIBA Jobin Yvon FL3-211 fluorescence spectrometer. The FT-IR was performed on Thermo Fisher Scientific's NicoletiS50 spectrometer. The gas adsorption and desorption experiments were carried out on a fully automated high-precision specific surface area and pore size microporous analyser, BSD-660M A3M, manufactured by Best Instrument Technology (Beijing) Co. TGA was performed on a Netzsch TGA209F1 instrument (Germany) under a nitrogen atmosphere at a heating rate of 10 ℃ min^{-1} from 25 to 800 ℃. Energy-dispersive X-ray spectroscopy (EDS) elemental mapping was performed on a ZEISS Sigma 300 FE-SEM equipped with an EDS detector to characterize the elemental distribution of In-TBAPy powder samples. Fluorescence measurements were performed on a Quanta Master 8000 spectrofluorometer. To assess the selectivity of In-TBAPy against interferents, each interferent (KCl, $MgCl_2$, NaCl, $CaCl_2$, glycine, proline, glucose, and urea) was added at 7.4×10^{-4}M, and the fluorescence intensity at the maximum emission wavelength was recorded.

1.2. Synthesis of In-TBAPy

0.0220 g (0.1 mmol) of indium nitrate pentahydrate ($In(NO_3)_3 \cdot 5H_2O$) and 0.0138 g (0.1 mmol) of H_4TBAPy were weighed and added into the solvent system of deionised water and N,N-dimethylformamide (DMF) in the volumes of 4 mL and 8 mL, respectively, and then 4 mL of 1-4 dioxane and 40 μL of concentrated hydrochloric acid were added into the mixture. Subsequently, 4 mL of 1-4 dioxane and 40 μL of concentrated hydrochloric acid were added to the mixed solution, and the solution was stirred thoroughly at room temperature to achieve homogeneous mixing, so as to obtain the mixed solution; then the obtained solution was transferred to a 50 mL reactor and placed in a constant-temperature drying oven, and the solvent was thermally reacted for 12 h at 85 ℃, and then naturally cooled down to room temperature; finally, the powder was washed with DMF and ethanol for 3 times, and then dried in vacuum, and the finely crushed light yellow powder was obtained. After vacuum drying, a fine yellowish powder In-TBAPy was obtained.

1.3. Fluorescence sensing experiments

For fluorescence response testing, 10 mg of In-TBAPy powder sample was accurately weighed into a glass reagent bottle containing 100 mL of deionised water. The powder was ultrasonically dispersed for 10 min in an ultrasonic processor to form a stable, homogeneous suspension. 2 mL of the suspension was injected into a four-sided translucent quartz cuvette, and then trace amounts of low-solubility deionised water solutions of various analytes were gradually added to the cuvette for fluorescence sensing experiments. During the experiment, the steady-state emission spectra of various target analytes at different concentration gradients were collected based on a fluorescence spectrometer to obtain the data matrices of training samples and blind test samples for subsequent data processing.

1.4. Limit of Detection (LOD)

Limit of Detection (LOD) refers to the minimum concentration or amount of analyte to be measured that can be reliably detected.

LOD refers to the minimum concentration or amount of the analyte to be detected reliably, which is an important index to characterise the sensing performance of the material. The initial fluorescence intensity of the In-TBAPy aqueous suspension was first recorded by fluorescence spectroscopy. Subsequently, standard aqueous solutions of different concentration gradients of analytes were added dropwise to the In-TBAPy aqueous suspension at the same excitation wavelength, and the corresponding fluorescence spectral data were recorded. After obtaining the initial data, the standard deviation Sb of the blank group was first calculated to characterise the degree of fluctuation of the material, and the standard deviation Sb can be calculated from equation 1:

$$S_b = \sqrt{\frac{\Sigma(I_0 - I_a)^2}{N-1}}$$

where I_0 is the initial fluorescence intensity of the In-TBAPy aqueous suspension, I_a is the mean value of I_0, and N is the number of times (usually 10) that the initial fluorescence intensity of the InTBAPy aqueous suspension was tested.

The LOD can be calculated from equation 2:

$$LOD = \frac{3 \times S_b}{S}$$

where S is the slope of the fitted curve and S_b is the standard deviation of the blank group.

1.5. Linear Discriminant Analysis

Linear Discriminant Analysis (LDA) is a classical data reduction and classification algorithm. It is mainly applied to supervised learning tasks, aiming to project high-dimensional data into a low-dimensional space, while maximising the inter-class distance and minimising the intra-class distance, in order to achieve the purpose of extracting and classifying features of experimental data. The steps of using linear discriminant analysis in this paper are as follows:

First, the steady-state fluorescence spectral data of In-TBAPy before and after the addition of the four neurotransmitters were collected. The intensities of the individual fluorescence spectra were also quantified in preparation for subsequent data analysis.

Next, for the change of fluorescence emission intensity of the same analyte at different concentrations, normalisation was performed, and the four luminescence sensing signals, I-440 nm, I-455 nm, I-475 nm and I-510 nm, were used to classify and differentiate,

and the fluorescence emission intensity (I) at each concentration was divided by the intrinsic fluorescence intensity (I_0) corresponding to the initial concentration, to obtain I/I_0, for the subsequent analysis and comparison. analysis and comparison.

After obtaining the normalized fluorescence intensity data, the above normalized data were imported into the JMP Pro 16 statistical modeling platform, and the discriminant function model was constructed by the LDA algorithm. Subsequently, typical discriminant maps were generated to identify and quantitatively analyse the four neurotransmitters. In this process, linear discriminant analysis can effectively distinguish the fluorescence response characteristics of different neurotransmitters. In addition, importing the normalised fluorescence intensity data of multivariate mixtures can also achieve the differentiation of multivariate mixtures.

1.6. Quantitative Fitting of Fluorescence Titration Curves

(1) Stern-Volmer model for 5-HT quenching system

$$\frac{I_0}{I} = 1 + K_{SV}[M]$$

I_0: initial fluorescence intensity; I: intensity after adding analyte; $[M]$: molar concentration of neurotransmitter; K_{SV}: Stern-Volmer quenching constant (M^{-1}).

[1] Benesi-Hildebrand double-reciprocal model for DA, A, NA fluorescence turn-on system

$$\frac{1}{\Delta I} = \frac{K_d}{\Delta I_{max}[G]} + \frac{1}{\Delta I_{max}}$$

I: fluorescence intensity change at 440 nm; ΔI_{max}: maximum fluorescence change; [G]: guest concentration; Kd: apparent dissociation constant (M).

2. Supporting Figures and Tables

2.1. Supporting Figures

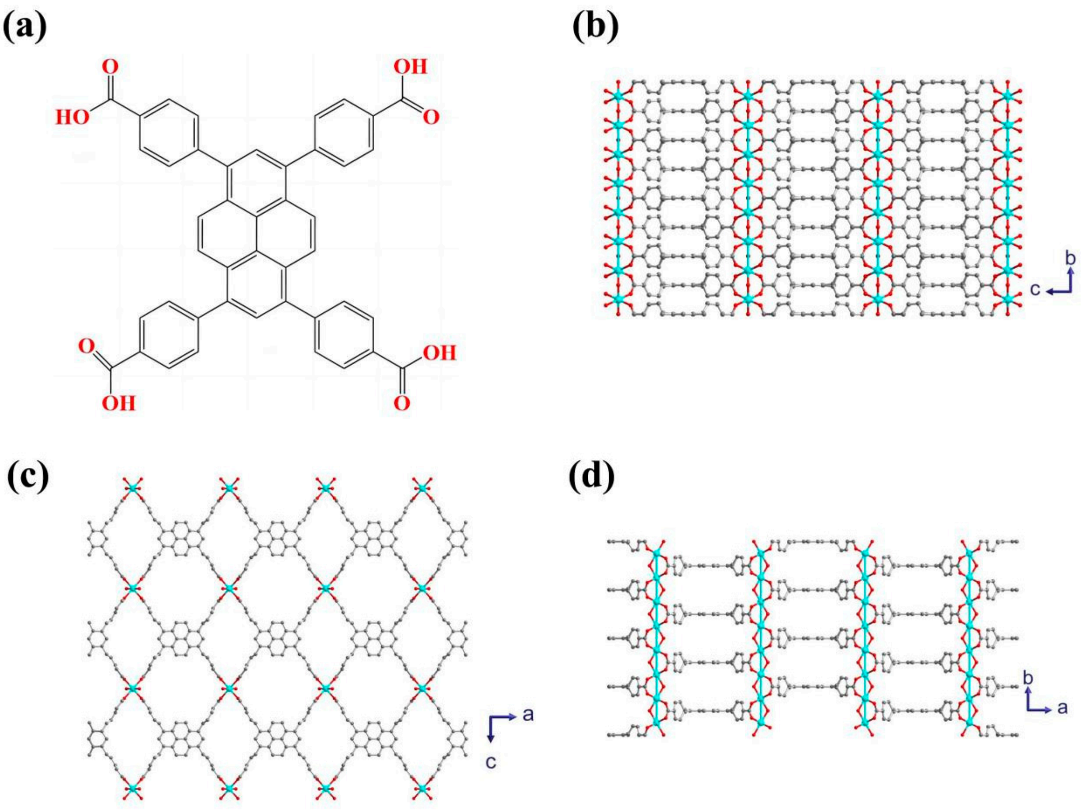

Figure S1. (a) Structure of H4BPTC; pore structure of In-TBAPy along (b) a-axis, (c) b-axis and (d) c-axis directions.

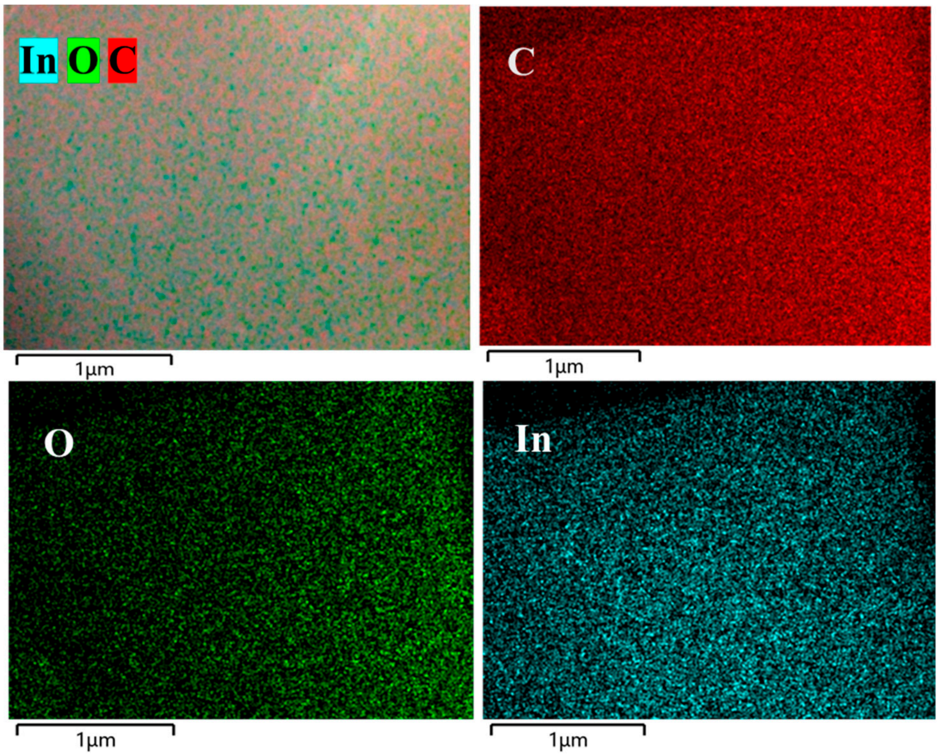

Figure S2. Energy-dispersive X-ray spectroscopy (EDX) elemental mapping of indium (In), carbon (C) and oxygen (O) of as-synthesized In-TBAPy.

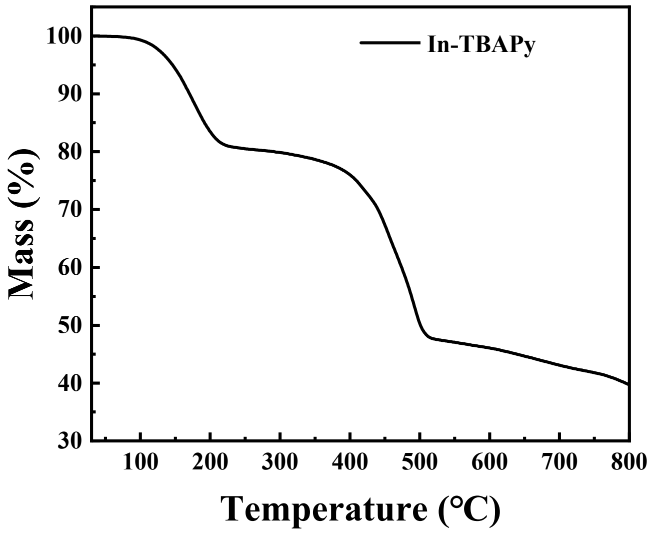

Figure S3. TGA curve of as-synthesized In-TBAPy.

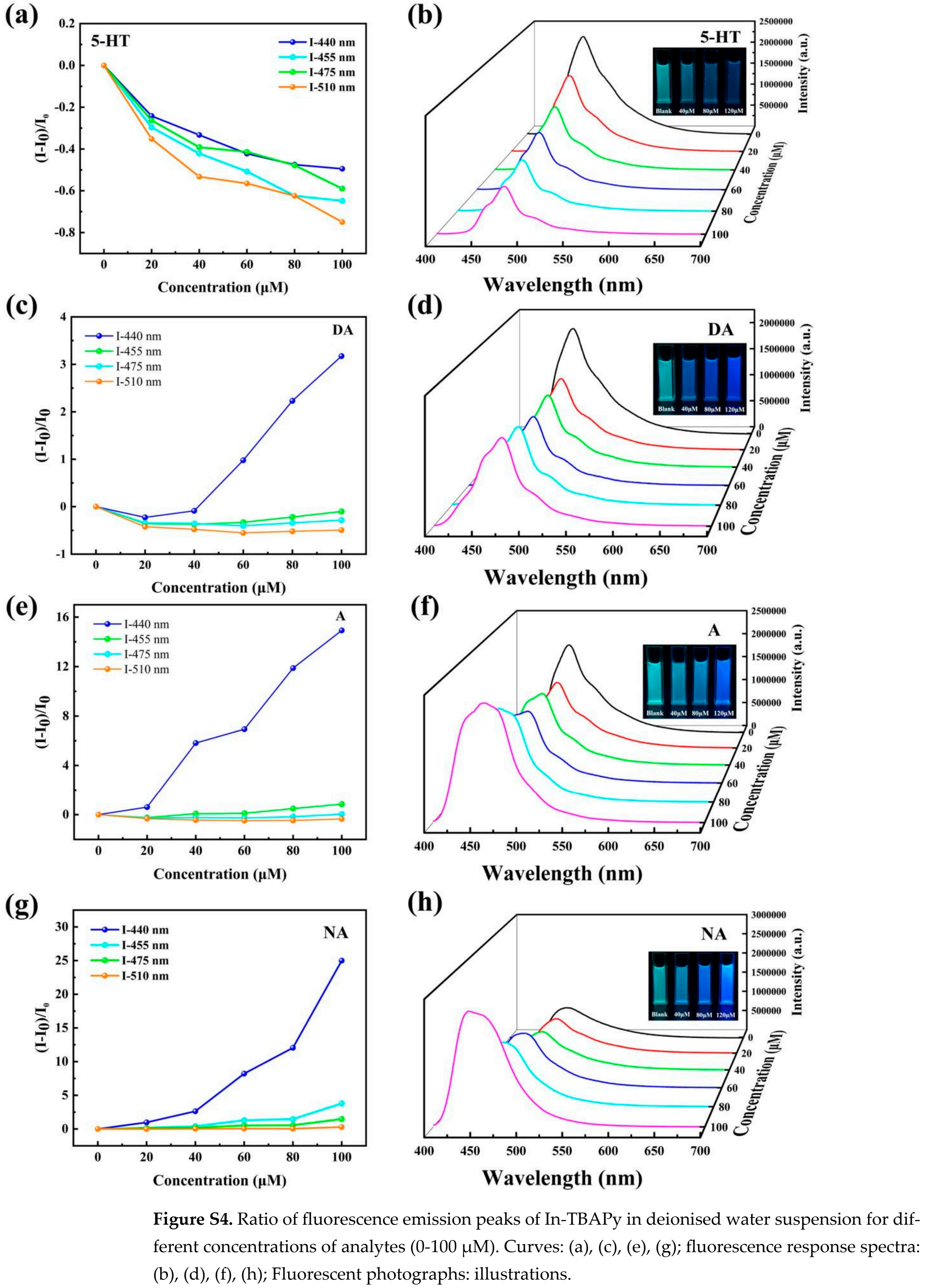

Figure S4. Ratio of fluorescence emission peaks of In-TBAPy in deionised water suspension for different concentrations of analytes (0-100 μM). Curves: (a), (c), (e), (g); fluorescence response spectra: (b), (d), (f), (h); Fluorescent photographs: illustrations.

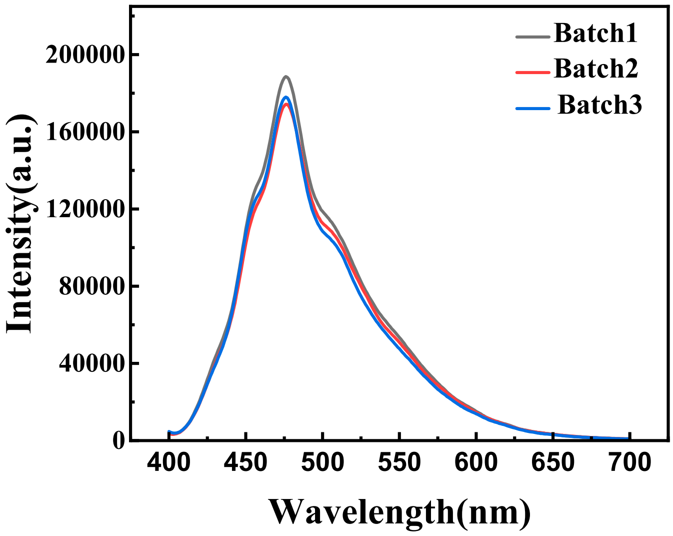

Figure S5. Fluorescence spectra of aqueous suspensions of three freshly prepared In-TBAPy batches without neurotransmitters.

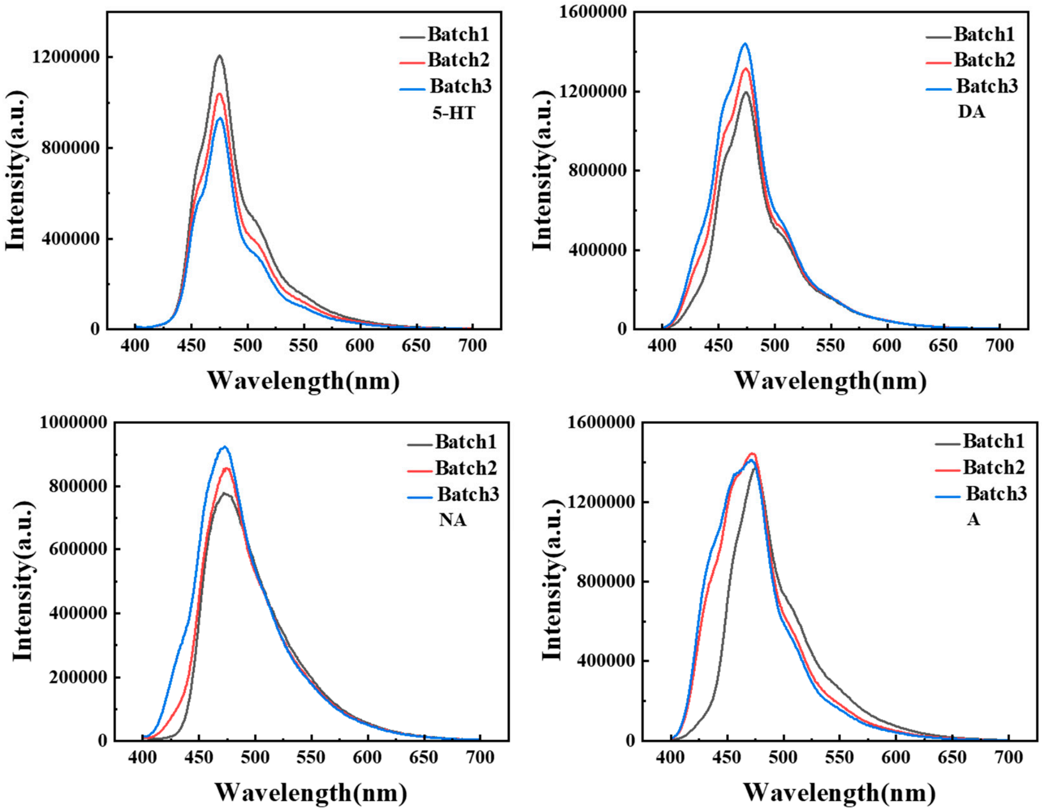

Figure S6. Fluorescence response spectra of aqueous suspensions of three independent In-TBAPy batches after incubation with four neurotransmitters (60μM). (a) 5-HT; (b) DA; (c) NA; (d) A.

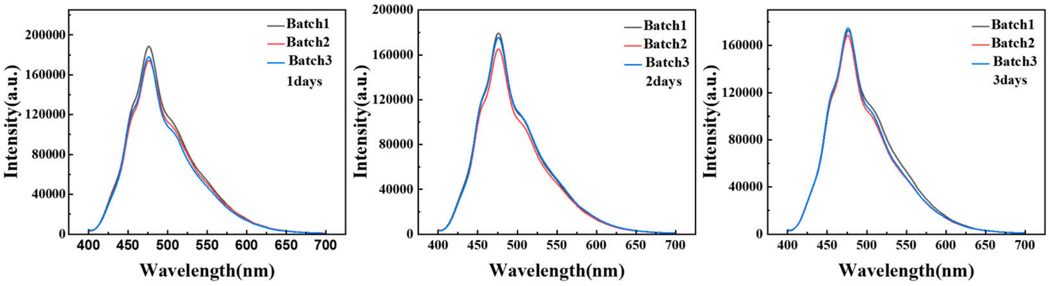

Figure S7. Intrinsic aqueous fluorescence spectra of three In-TBAPy batches after being sealed and stored for different durations. (a) Stored for 1 day; (b) Stored for 2 days; (c) Stored for 3 days.

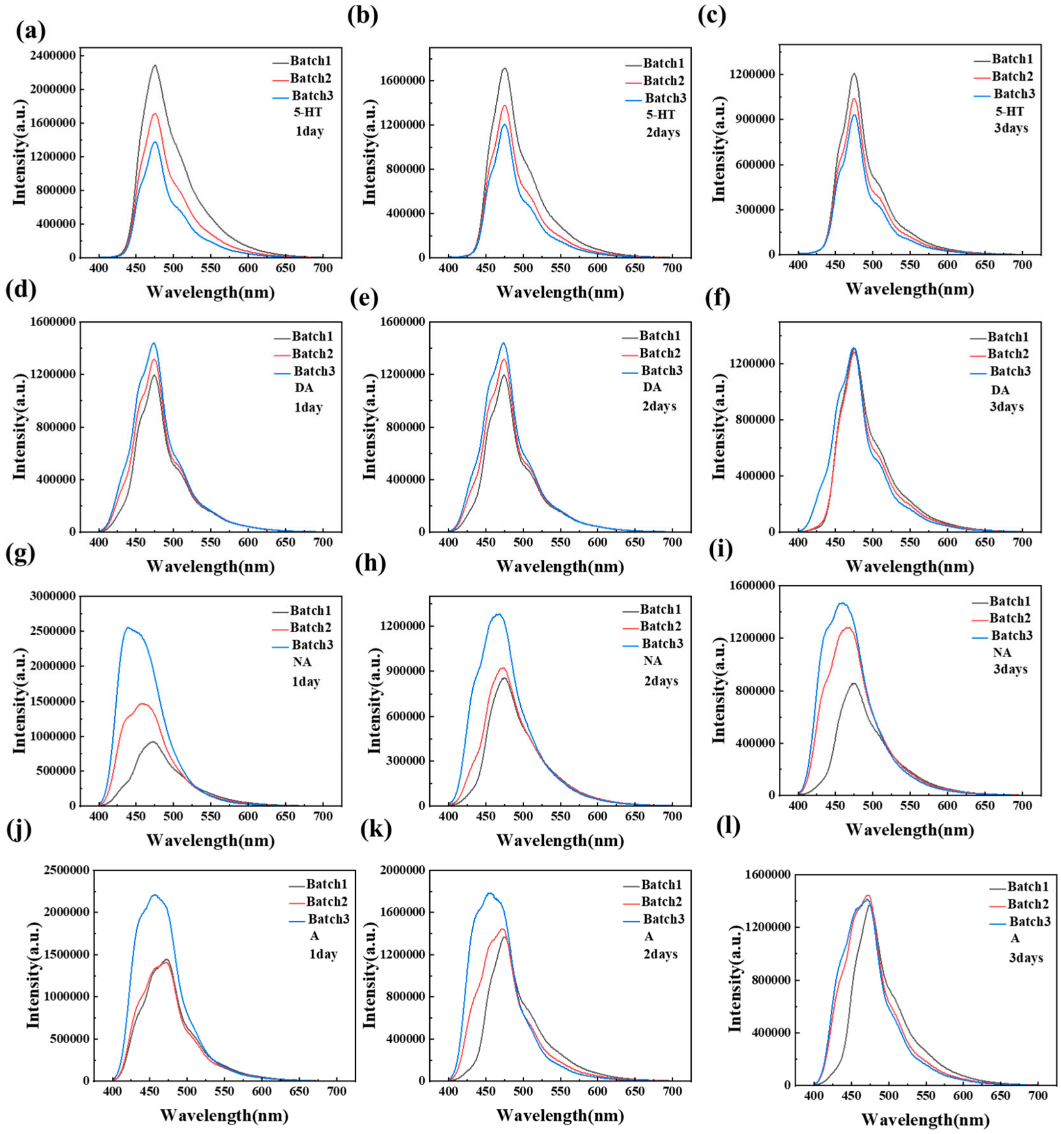

Figure S8. Fluorescence response spectra of three In-TBAPy batches after being sealed and stored for 1, 2 and 3 days, upon reaction with four neurotransmitters(60μM). (a-c) 5-HT after storage for 1, 2, 3 days; (d-f) DA after storage for 1, 2, 3 days; (g-i) NA after storage for 1, 2, 3 days; (j-l) A after storage for 1, 2, 3 days.

.

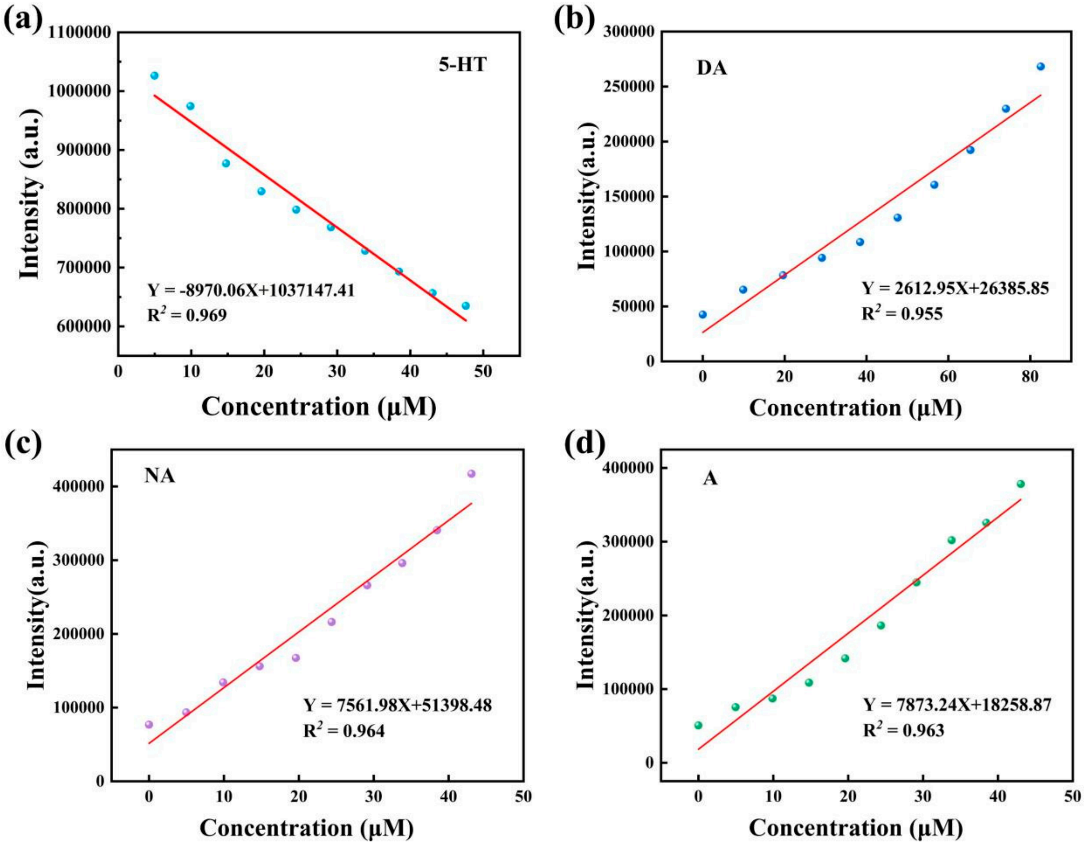

Figure S9. Linear relationship between the luminous intensity of In-TBAPy and the concentrations of (a) 5-HT; (b) DA; (c) A and (d) NA.

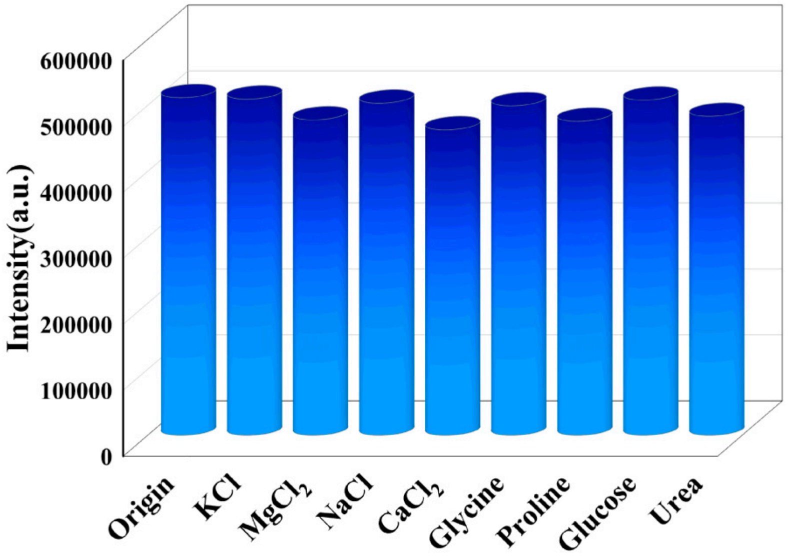

`

Figure S10. Fluorescence response of In-TBAPy to various interferents.

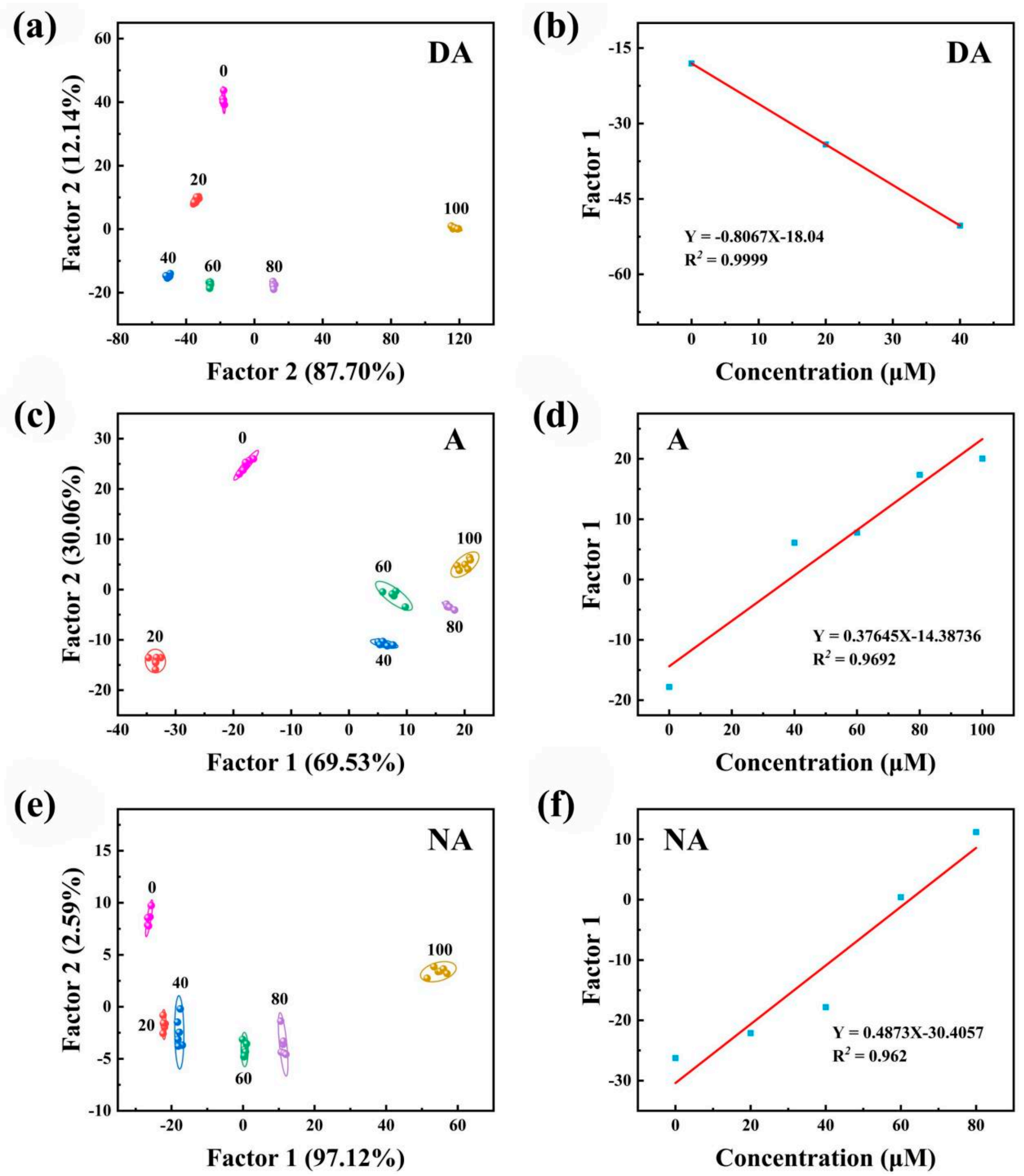

Figure S11. Typical plots of fluorescence intensity response data of In-TBAPy in deionised water suspension to different concentrations of analytes (0-100 μM) obtained after linear discriminant analysis: (a), (c), (e) and linear fitting plots differentiated by the first typical factor: (b), (d), (f).

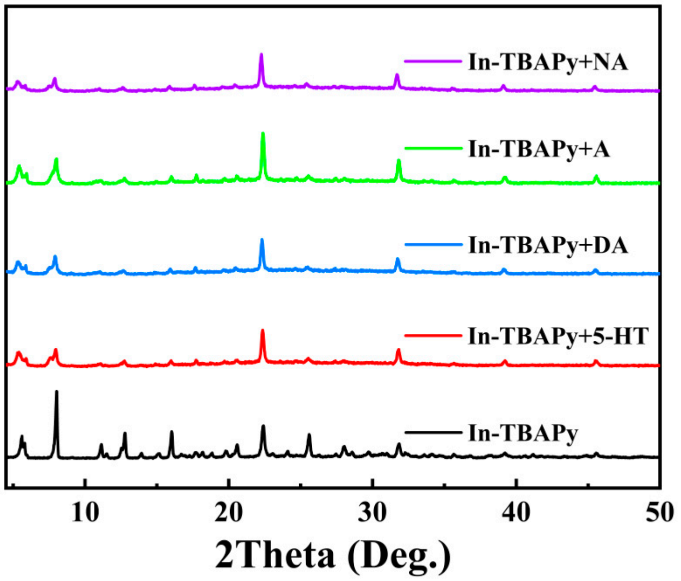

Figure S12. PXRD spectra of In-TBAPy before and after immersion in four neurotransmitter biomarkers at a concentration of 100 μM.

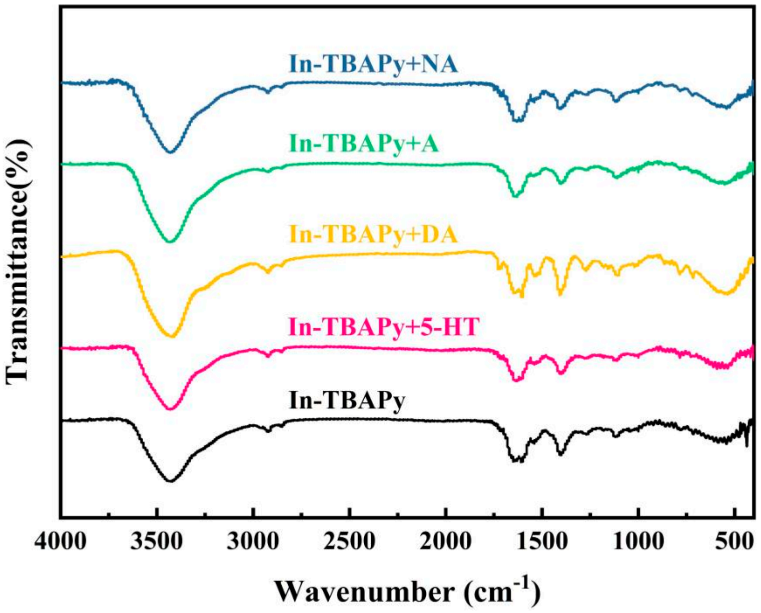

Figure S13. FT-IR spectra of In-TBAPy before and after immersion in four neurotransmitter biomarkers at a concentration of 100 μM.

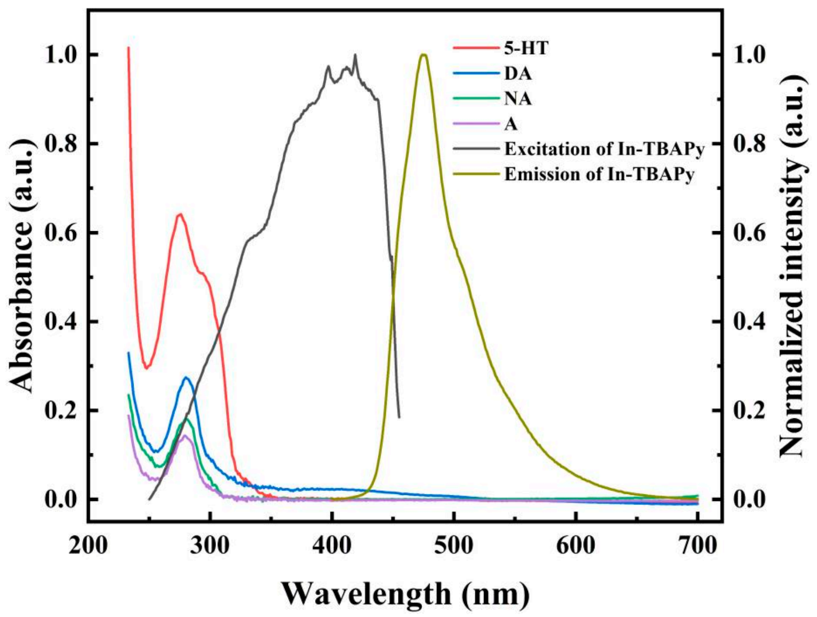

Figure S14. UV-visible absorption profile with In-TBAPy excitation and emission profiles.

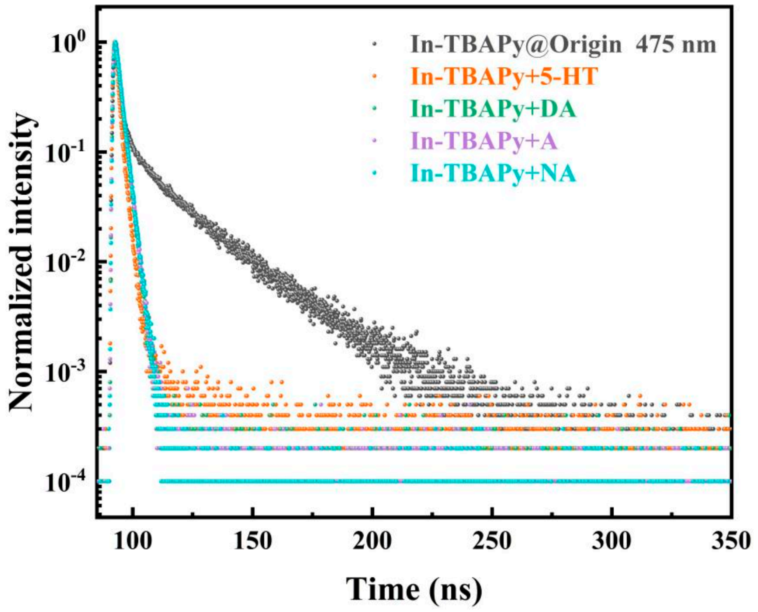

Figure S15. Fluorescence decay life curve at 475 nm.

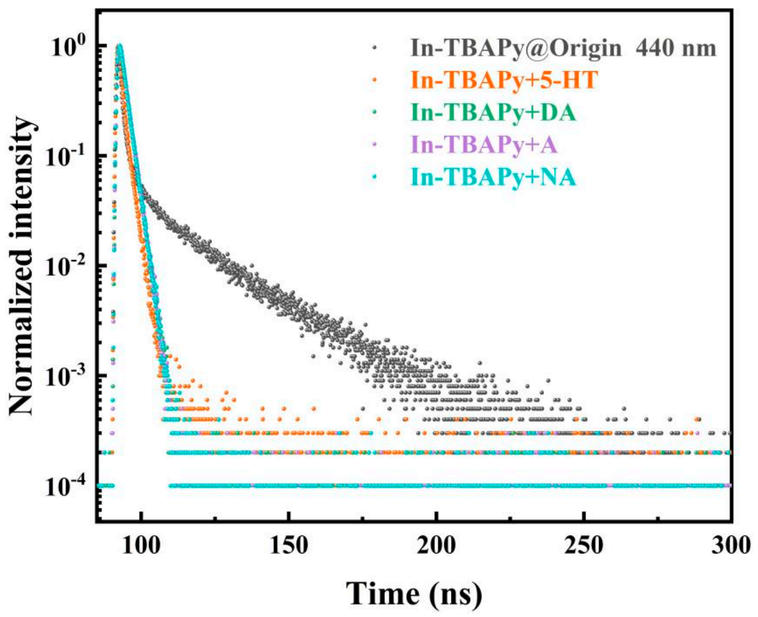

Figure S16. Fluorescence decay life curve at 440 nm.

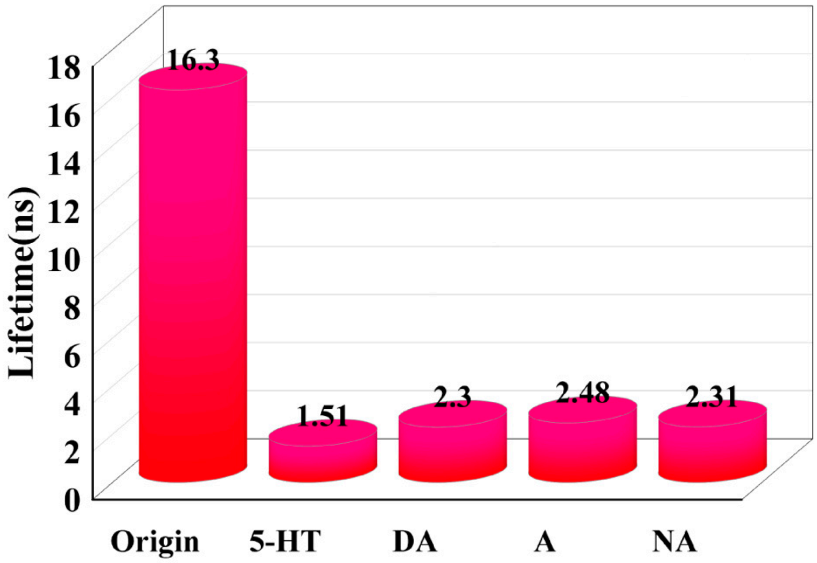

Figure S17. Fluorescence lifetime at 440 nm after fitting.

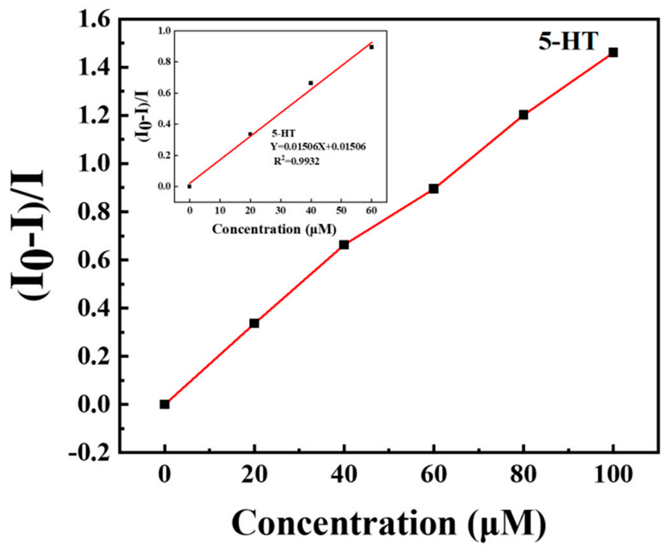

Figure S18. Stern-Volmer linear fitting curve of In-TBAPy upon serial addition of 5-HT at 475 nm. Inset shows fitting equation.

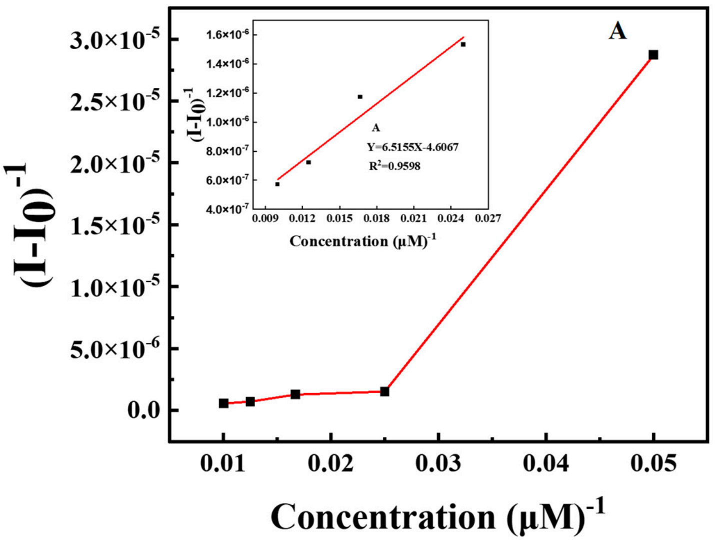

Figure S19. Benesi-Hildebrand double-reciprocal fitting curve of A fluorescence response at 440 nm for calculating apparent K_d Inset shows fitting equation.

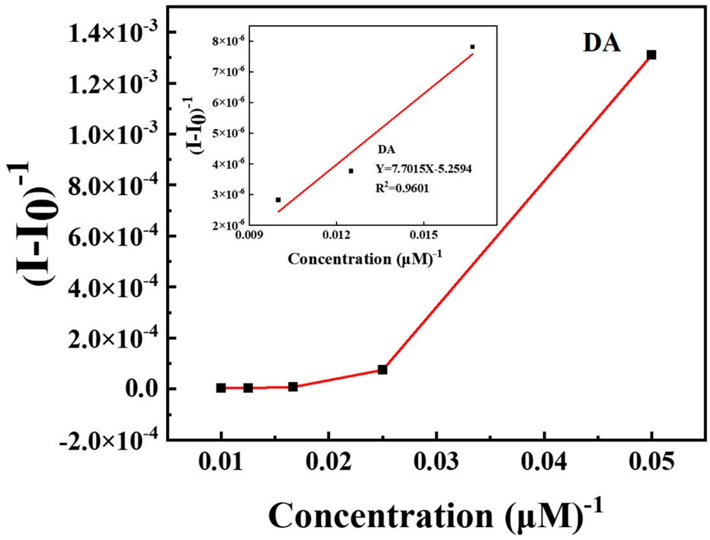

Figure S20. Benesi-Hildebrand double-reciprocal fitting curve of DA fluorescence response at 440 nm for calculating apparent K_d Inset shows fitting equation.

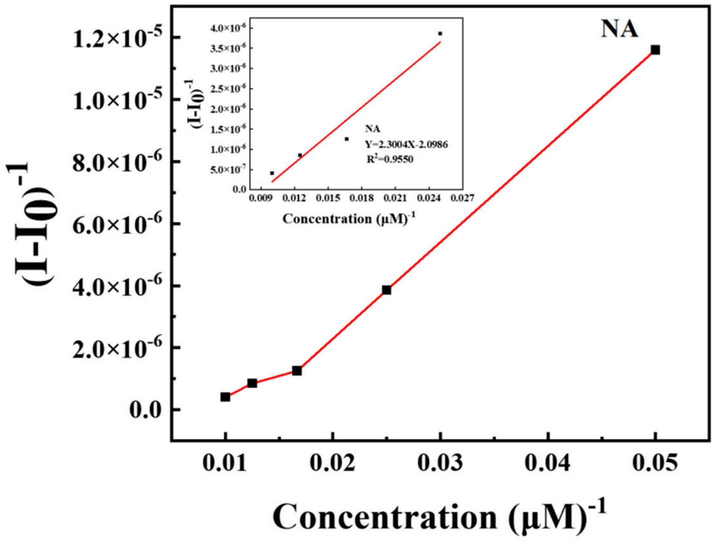

Figure S21. Benesi-Hildebrand double-reciprocal fitting curve of NA fluorescence response at 440 nm for calculating apparent K_d Inset shows fitting equation.

2.2. Supporting Tables

Table S1. Blind test response and prediction of In-TBAPy for four analytes at 60 μM.

Blind samples	440 nm	455 nm	475 nm	510 nm	Predication
5-HT	-0.33145	-0.41807	-0.38608	-0.53731	5-HT
5-HT	-0.33973	-0.42555	-0.40228	-0.55062	5-HT
5-HT	-0.5107	-0.51073	-0.4802	-0.63036	5-HT
5-HT	-0.32155	-0.42007	-0.39008	-0.54031	5-HT
5-HT	-0.32973	-0.42455	-0.40228	-0.54862	5-HT
5-HT	-0.5007	-0.50973	-0.4762	-0.62936	5-HT
5-HT	-0.49486	-0.50525	-0.47456	-0.62298	5-HT
5-HT	-0.49189	-0.50593	-0.48077	-0.62256	5-HT
5-HT	-0.49109	-0.50492	-0.47649	-0.62544	5-HT
5-HT	-0.49527	-0.51361	-0.48116	-0.62241	5-HT
5-HT	-0.48263	-0.56928	-0.54284	-0.6985	5-HT
5-HT	-0.44584	-0.39039	-0.38519	-0.5409	5-HT
5-HT	-0.47323	-0.38504	-0.37014	-0.5306	5-HT
5-HT	-0.4827	-0.41688	-0.37893	-0.52648	5-HT
5-HT	-0.48917	5.004871	-0.39246	-0.52827	5-HT
5-HT	-0.41795	-0.62322	-0.58895	-0.75095	5-HT
5-HT	-0.43768	-0.62109	-0.58791	-0.75047	5-HT
5-HT	-0.41611	-0.62323	-0.59354	-0.74624	5-HT
5-HT	-0.42117	-0.62408	-0.58631	-0.75245	5-HT
5-HT	-0.41881	-0.6292	-0.59274	-0.74798	5-HT
DA	16.80037	2.737118	1.054541	-0.10619	DA
DA	18.61523	2.902017	1.053995	-0.10549	DA
DA	20.0239	3.090155	1.107385	-0.10774	DA
DA	19.03301	3.002671	1.056992	-0.06697	DA
DA	0.770224	-0.33307	-0.45718	-0.58783	A
DA	2.630786	0.109429	-0.2203	-0.54796	5-HT
DA	2.806384	0.105687	-0.20887	-0.54291	5-HT
DA	8.446397	1.519584	0.38097	-0.23019	DA
DA	3.699685	0.132204	-0.22169	-0.57777	5-HT
DA	3.545029	0.173956	-0.23456	-0.57099	5-HT
DA	6.577116	0.86587	0.088378	-0.45908	DA
DA	6.862725	1.320805	0.375522	-0.20431	DA
DA	8.129958	1.365937	0.3974	-0.20544	DA
DA	8.64415	1.338874	0.348206	-0.23364	DA
DA	8.335397	1.409584	0.37697	-0.22919	DA
DA	15.54272	2.810258	1.057022	-0.06196	DA
DA	15.77737	2.737118	1.054541	-0.05619	DA
DA	18.61523	2.962017	1.053995	-0.07549	DA
DA	19.9239	2.889155	1.087385	-0.08774	DA
DA	19.03291	3.00267	1.056992	-0.06697	DA
A	5.097088	0.483841	-0.00981	-0.35086	A
A	5.262302	0.49455	-0.03236	-0.37342	A
A	4.952585	0.482868	-0.00889	-0.34773	A
A	5.107221	0.508479	-0.00923	-0.3555	A
A	-0.19637	-0.47197	-0.46833	-0.51411	A
A	-0.11226	-0.43043	-0.45158	-0.5201	A
A	-0.07582	-0.4275	-0.4608	-0.51588	A
A	-0.08571	-0.42695	-0.46105	-0.5205	A
A	-0.07472	-0.42475	-0.4694	-0.53688	A
A	4.021924	0.145803	-0.25858	-0.54705	A
A	4.354284	0.182736	-0.25522	-0.54536	A
A	4.407089	0.203284	-0.27534	-0.5537	A
A	4.596791	0.234453	-0.25465	-0.54997	A
A	4.747642	0.26898	-0.23965	-0.54486	A
A	4.148239	0.234156	-0.15183	-0.45853	A
A	3.773051	0.227447	-0.15234	-0.40965	A
A	4.067912	0.264343	-0.13822	-0.41906	A
A	4.147559	0.289656	-0.11385	-0.39591	A
A	4.072056	0.303827	-0.11932	-0.3993	A
A	4.829862	0.351494	-0.11874	-0.46678	A

NA	0.811178	0.090976	0.106398	-0.00762	NA
NA	0.930129	0.140986	0.158418	0.026789	NA
NA	0.945157	0.189931	0.173816	0.018682	NA
NA	1.213006	0.219545	0.121851	-0.03906	NA
NA	1.016426	0.191898	0.141085	-0.01037	NA
NA	2.4398	0.362546	0.184459	-0.00477	NA
NA	2.553456	0.384842	0.242132	0.016931	NA
NA	2.645614	0.437125	0.196321	-0.02669	NA
NA	2.992819	0.403009	0.16244	-0.0709	NA
NA	2.515595	0.393024	0.193167	-0.0239	NA
NA	7.511594	1.243484	0.578162	0.087874	NA
NA	7.769793	1.205422	0.544059	0.071754	NA

**Blind samples**	**440 nm**	**455 nm**	**475 nm**	**510 nm**	**Predication**
NA	7.988754	1.314086	0.579754	0.06103	NA
NA	9.543426	1.406592	0.524863	0.006312	NA
NA	8.247092	1.317023	0.535626	0.028187	NA
NA	11.05769	1.703678	0.690658	0.060552	NA
NA	11.54483	1.362212	0.438337	0.030018	NA
NA	11.85334	1.399825	0.477742	0.036441	NA
NA	13.68777	1.380399	0.467056	0.026974	NA
NA	12.11848	1.392889	0.492132	0.0458	NA

Table S2. Confusion matrix of training set classification based on 80 samples.

**Actual \ Predicted**	**5-HT**	**A**	**DA**	**NA**	**Total**	**Correct**
5-HT	20	0	0	0	20	20
A	0	20	0	0	20	20
DA	4	1	15	0	20	15
NA	0	0	0	20	20	20

Table S3. Comparison of In-TBAPy MOF array with other MOF-based sensors.

**Sensor Material**	**Sensing Type**	**Analytes**	**Linear Range**	**Detection Limit (LOD)**	**Ref**
PFC-1 (HOF)	Ratiometric fluorescence	5-HT, NE, Adr, DA	1.0–14.8 μM (low concentration)	5-HT: 36 nM; Adr: 157 nM; NE: 263 nM; DA: 757 nM	[1]
HOF-BTB-NH_2	Fluorescence turn-off	5-HT	0.5–38.5 μM	1.21 μM	[2]
Eu^{3+}/Tb^{3+}@UiO-66-$(COOH)_2$/NDC	Dual-emission ratiometric fluorescence	Three nitrophenol isomers:	40–100μM	—	[3]
ZJU-235 / ZJU-235-NH_2 / ZJU-236 (MOF array)	Multi-channel fluorescence fingerprint	Eight metal ions	20–100 μM	—	[3]
HOF-TCBP	Fluorescence turn-off	DA	0–50 μM	36.57 μM	[4]
ACF@Eu-MOF	Ratiometric fluorescence turn-off	5-HT	0–28 μM	86 nM	[5]
HOF-DBA	Fluorescence turn-off	Nitrofurazone	10^{-9} – 10^{-3} M	5.55 nM	[6]
HOF-DBA	Fluorescence turn-on	γ-Aminobutyric acid	10^{-8} – 10^{-3} M	58.2 nM (aqueous) / 126 nM (serum)	[6]
UiO-66-NH_2 MOF	Ratiometric fluorescence	DA	4–50 μM	0.68 μM	[7]
In-TBAPy	Fluorescence array	5-HT, DA, A, NA	5-HT/A: 0–100 μM; DA: 0–40 μM; NA: 0–80 μM	5-HT: 2.26 μM; DA: 10.13 μM; A: 3.50 μM; NA: 3.36 μM	**This work**

References

[1] W. Zhang, Z. Zhang, G. Bai, S. Xu, H. Xu, J. Gao, Excimer-to-monomer switching in a pyrene HOF for discriminative neurotransmitter sensing, Chemical Communications, **2026**, 62, 1510–1514.

[2] F. Chen, Y. Cai, P. Shen, G. Bai, T. Alshahrani, J. Gao, B. Chen, S. Xu, H. Xu, A porous hydrogen-bonded organic framework for sensitive and highly selective fluorescence sensing of carcinoid biomarkers, Microporous and Mesoporous Materials, **2023**, 361, 112759.

[3] Z. Jiang, H.-Q. Zheng, L. Guan, Y. Yang, Y. Cui, G. Qian, Enhanced luminescence in multivariate metal–organic frameworks through an isolated-ligand strategy. Journal of Materials Chemistry C, **2022**, 10, 10473–10479.

[4] L.-M. Dai, L. Li, E.-L. Wang, S.-Y. You, L. Zhang, Z. Gu, M.-F. Wu, J.-Y. Zou, Encapsulation of Fluorophores within the Lanthanide Metal–Organic Framework for Regulating the Ratiometric Dynamic Luminescence Performance of Serotonin Detection. Inorganic Chemistry, **2025**, 64, 13775–13785.

[5] K.-Y. Wu, M. Chen, N.-H. Huang, R.-T. Li, W.-L. Pan, W.-H. Zhang, W.-H. Chen, J.-X. Chen, Facile and recyclable dopamine sensing by a label-free terbium(III) metal−organic framework, Talanta, **2021**, 221, 121399.

[6] Y. Liu, X. Xu, B. Yan, An anthracene-based hydrogen-bonded organic framework as a bifunctional fluorescent sensor for the detection of γ-aminobutyric acid and nitrofurazone, Inorganic Chemistry Frontiers, **2022**, 9, 3627–3635.

[7] N. Wang, M. Xie, M. Wang, Z. Li, X. Su, UiO-66-NH2 MOF-based ratiometric fluorescent probe for the detection of dopamine and reduced glutathione, Talanta, **2020**, 220, 121352.
